# Efficacy and Safety of Oral Factor XIa Inhibitors in Stroke Prevention: A Systematic Review and Meta-Analysis

**DOI:** 10.3390/jcm12175562

**Published:** 2023-08-26

**Authors:** Lina Palaiodimou, Georgia Papagiannopoulou, Aristeidis H. Katsanos, Andreas Eleftheriou, Theodore Karapanayiotides, Panayiotis D. Mitsias, Robin Lemmens, Carlos A. Molina, Andrei Alexandrov, Valeria Caso, Ashkan Shoamanesh, Mukul Sharma, Georgios Tsivgoulis

**Affiliations:** 1 Second Department of Neurology, “Attikon” University Hospital, School of Medicine, National and Kapodistrian University of Athens, 12462 Athens, Greece; 2 Division of Neurology, McMaster University/Population Health Research Institute, Hamilton, ON L8L2X2, Canada; 3 Second Department of Neurology, School of Medicine, Faculty of Health Sciences, AHEPA University General Hospital, Aristotle University of Thessaloniki, 54636 Thessaloniki, Greece; 4 Neurology Department, University General Hospital of Heraklion, 71500 Heraklion, Greece; 5 Experimental Neurology, Department of Neurosciences, Katholieke Universiteit Leuven, 3000 Leuven, Belgium; 6 VIB, Center for Brain & Disease Research, Laboratory of Neurobiology, 3001 Leuven, Belgium; 7 Department of Neurology, University Hospitals Leuven, 3000 Leuven, Belgium; 8 Vall d’Hebron Stroke Center, Department of Neurology, Hospital Universitari Vall d’Hebron, Departament de Medicina, Universitat Autònoma de Barcelona, 08035 Barcelona, Spain; 9 Department of Neurology, University of Tennessee Health Science Center, Memphis, TN 38163, USA; 10 Stroke Unit, Santa Maria della Misericordia Hospital, University of Perugia, 06129 Perugia, Italy

**Keywords:** ischemic stroke, stroke prevention, antithrombotic treatment, anticoagulation, factor Xia inhibitors, meta-analysis, randomized-controlled clinical trials

## Abstract

Introduction: Despite preventive measures, stroke rates remain high in the primary and secondary prevention settings. Factor XIa inhibition may offer a novel, safe and effective antithrombotic option for stroke prevention. Methods: We conducted a systematic review and meta-analysis including all available randomized controlled clinical trials (RCTs) that investigated the efficacy and safety of factor XIa inhibitors versus controls in primary or secondary stroke prevention. The primary efficacy and safety outcomes of interest were symptomatic ischemic stroke (IS) and the composite of major bleeding and clinically relevant non-major bleeding. Results: Four phase II dose-finding RCTs were included, comprising a total of 4732 patients treated with factor XIa inhibitors versus 1798 controls. Treatment with factor XIa inhibitors did not reduce the risk of IS compared to controls (RR: 0.89; 95% CI: 0.67–1.17). The composite of symptomatic IS and covert infarcts on brain MRI (RR: 1.01; 95% CI: 0.87–1.18), the composite of symptomatic IS and transient ischemic attack (TIA; RR: 0.78; 95% CI: 0.61–1.01), and the composite of major adverse cardiovascular events (RR: 1.07; 95% CI: 0.87–1.31) did not differ between the treatment groups. Treatment with factor XIa inhibitors did not increase the risk of the composite of major bleeding and clinically relevant non-major bleeding (RR: 1.19; 95% CI: 0.65–2.16), major bleeding alone (RR: 1.19; 95% CI: 0.64–2.22), intracranial bleeding (RR: 0.91; 95% CI: 0.26–3.19) or all-cause mortality (RR: 1.21; 95% CI: 0.77–1.90). Conclusion: This meta-analysis provides reassuring evidence regarding the safety of factor XIa inhibitors. These findings, coupled with potential signals of efficacy in reducing IS (and TIA), underscore the importance of ongoing phase III RCTs for providing definitive data regarding the effect of factor XIa inhibition on stroke prevention.

## 1. Introduction

Stroke is the second-leading cause of death and the third-leading cause of death and disability combined worldwide [1]. Yet, stroke preventive measures are inadequate for a significant proportion of patients, both in primary and in secondary prevention settings [2]. Antithrombotic treatment is the cornerstone of ischemic stroke (IS) prevention, but the selection of appropriate antithrombotic regimens, alone or in combination, should respect the delicate balance between thrombosis and bleeding [3]. To this end, factor XIa inhibitors have been actively tested as a novel anticoagulant, aiming to prevent thrombotic events and stroke in high-risk patients without increasing (intracranial) bleeding risk [4,5,6,7].

With the inhibition of factor XIa, there is a theoretical potential to uncouple hemostasis and vascular thrombosis [8]. Through factor XIa inhibition, thrombin amplification is prohibited, which prevents the formation of pathological thrombi, while the tissue factor pathway continues to produce thrombin, which allows beneficial blood clots to form in order to produce hemostasis [9]. Evidence from in vivo animal models [10,11], patients with inherited factor XI deficiency [12], and randomized controlled clinical trials (RCTs) using antisense oligonucleotides [13], monoclonal antibodies [14,15], and small molecules targeting factor XIa [16] suggest that factor XIa inhibition may offer a safer anticoagulant option compared with previous generations of anticoagulants. Recently, a systematic review and meta-analysis summarizing the results of available RCTs comparing patients receiving factor XIa inhibitors versus controls did not detect any difference in hemorrhagic events between the two groups and confirmed the favorable safety profile of factor XIa inhibitors [17]. However, this study was limited by the inclusion of a quite heterogenous patient population (by merging patients at high risk for cardio- and cerebrovascular events [5,6,7] with those undergoing knee arthroplasty and requiring venous thromboembolism prevention [13,14,15,16]) and by the fact that it did not specifically assess stroke occurrence as an outcome of interest.

Considering the thus-far-unmet need to optimize stroke prevention while maintaining low rates of hemorrhagic adverse events, we specifically sought to investigate the efficacy and safety of factor XIa inhibitors in primary and secondary stroke prevention, using a meta-analysis of recently completed RCTs.

## 2. Methods

### 2.1. Standard Protocol Approvals, Registrations, and Patient Consent

The pre-specified protocol of the present systematic review and meta-analysis has been registered in the International Prospective Register of Ongoing Systematic Reviews PROSPERO (registration ID: CRD42022383298) and is reported according to the updated Preferred Reporting Items for Systematic Reviews and Meta-Analyses (PRISMA) guidelines [18]. No ethical board approval or written informed consent from the patients was required due to the study design (systematic review and meta-analysis).

### 2.2. Data Sources, Searches and Study Selection

Following the PICO format, a systematic literature search was conducted to identify available RCTs evaluating adult patients in either the primary or the secondary prevention setting (P: population), treated with any factor XIa inhibitor at any dose (I: intervention), versus placebo or standard of care (C: comparator). IS occurrence at follow-up (O: outcome) was required for studies to be considered eligible for inclusion. The literature search was performed independently by three reviewers (LP, GP, and AE). We searched MEDLINE, EMBASE, Scopus, and the Cochrane Library using search strings that included the following terms: “factor XIa inhibitor” and “stroke”; the complete search algorithms used are presented in the Supplementary Materials. No language or other restrictions were applied. Our search spanned from the inception of each database until 17 December 2022. We additionally searched reference lists of published articles and international conference abstracts manually, to ensure the comprehensiveness of the bibliography.

Observational studies, cohort studies, non-controlled studies, case series and case reports were excluded. Commentaries, editorials, and narrative reviews were also discarded. We also excluded RCTs not providing information on the primary efficacy outcome (IS occurrence). All retrieved studies were independently assessed by the three reviewers (LP, GP, and AE), and any disagreements were resolved after discussion with a fourth tie-breaking evaluator (GT).

### 2.3. Quality Control, Bias Assessment and Data Extraction

Eligible studies were subjected to quality control and bias assessment employing the Cochrane Collaboration tool (RoB 2) for RCTs [19]. Quality control and bias assessment was conducted independently by four reviewers (LP, GP, AHK, and AE), and disagreements were settled by consensus after discussion with the corresponding author (GT).

Data were predominantly extracted by scrutinizing the peer-reviewed publications of the included RCTs along with their Supplementary Materials. In the case of missing information, data were also sought in the presentations of the respective RCTs at international conferences. Data extraction was performed on structured forms, including author names, date of publication, country, inclusion criteria, patient sample, patients’ characteristics, and outcomes of interest.

### 2.4. Outcomes

The primary efficacy outcome of interest was the risk of symptomatic IS occurrence during follow-up among patients treated with factor XIa inhibitors versus controls.

Secondary efficacy outcomes of interest comprised the following: (i) the composite of symptomatic IS and covert brain infarction in the RCTs that included brain imaging at follow-up; (ii) the composite of symptomatic IS and transient ischemic attack (TIA); and (iii) major adverse cardiovascular events (MACE) as defined by the composite of cardiovascular death, systemic embolism, myocardial infarction and stroke.

The primary safety outcome of interest was the risk of the composite of major bleeding or clinically relevant non-major bleeding during follow-up among patients treated with factor XIa inhibitors versus controls.

Secondary safety outcomes of interest included the following: (i) major bleeding alone; (ii) intracranial hemorrhage; and (iii) all-cause mortality.

Both primary and secondary outcomes of interest were assessed in subgroup analyses stratified by (i) different settings (primary versus secondary stroke prevention), (ii) different factor XIa inhibitors (asundexian versus milvexian), and (iii) different experimental arms (factor XIa inhibitors plus standard of care vs. factor XIa inhibitors alone). We also conducted a sensitivity analysis in order to assess for a potential dose effect on the primary efficacy and safety outcomes. For this sensitivity analysis, the asundexian doses of 10 mg qd, 20 mg qd, and 50 mg qd were considered to be low, intermediate, and high doses of factor XIa inhibitor, respectively. In the case of AXIOMATIC-SSP and milvexian [4], doses of 25 mg qd and 25 mg bid were grouped together as low doses, the 50 mg bid dose was considered an intermediate dose, and doses of 100 mg bid and 200 mg bid were grouped together as high doses. High doses and intermediate doses were compared to the low doses, which were considered the reference group for this analysis. Due to testing only intermediate and high doses of asundexian, PACIFIC-AF [5] was excluded from this analysis.

### 2.5. Statistical Analysis

For the pairwise meta-analysis, we calculated for each dichotomous outcome of interest the corresponding risk ratios (RR) with 95% confidence interval (95% CI) for the comparison of outcome events among patients receiving factor XIa inhibitors versus controls. The random effects model (DerSimonian and Laird) was used to calculate the pooled estimates [20]. Subgroup differences between different subgroups were assessed using the Q test for subgroups [21]. Heterogeneity was assessed with the I^2^ and Cochran Q statistics. For the qualitative interpretation of heterogeneity, I^2^ values > 50% and values > 75% were considered to represent substantial and considerable heterogeneity, respectively. The significance level for the Q statistic was set at 0.1. Publication bias across individual studies was assessed when more than four studies were included in the analysis of the outcomes of interest, using both funnel plot inspection and the Egger’s linear regression test [22], and the equivalent z test for each pooled estimate with a two-tailed *p* value < 0.05 was considered statistically significant. Furthermore, the fragility index (FI) was calculated for the dichotomous outcomes of interest [23] and assessed based on the classification by Mun et al., suggesting that FI ≤ 4 was indicative of a “highly fragile/non-robust” result; 4 < FI ≤ 12 pointed to a “fragile/somewhat robust” result; 12 < FI ≤ 34 corresponded to a “robust” result; and, finally, FI > 34 was suggestive of a “highly robust” result [24]. All statistical analyses were performed using the Cochrane Collaboration’s Review Manager (RevMan 5.3) Software Package (Copenhagen: The Nordic Cochrane Centre, The Cochrane Collaboration, 2014) [25], and the OpenMetaAnalyst [26].

## 3. Results

### 3.1. Literature Search and Included Studies

**Figure 1:**
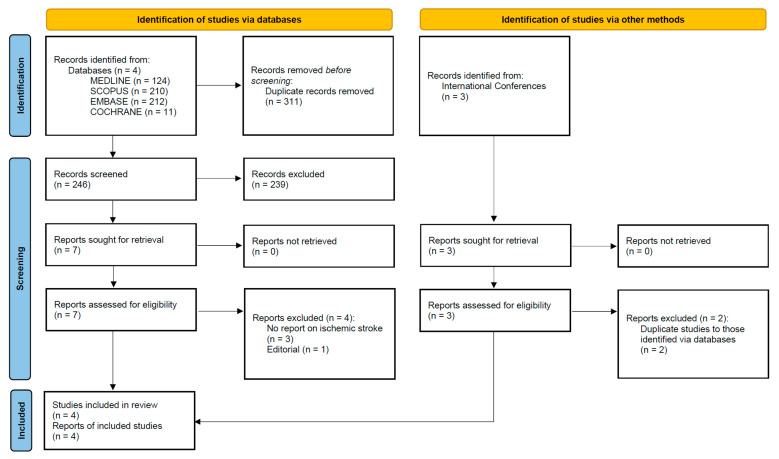
Flowchart of the systematic review.

After excluding duplicates, the systematic database search yielded a total of 246 records from the MEDLINE, EMBASE, SCOPUS, and Cochrane Library databases, while three studies were identified through a search of international conference abstracts (Figure 1). Following the initial screening of the studies identified via the search of the databases, we retrieved the full text of seven records that were considered potentially eligible for inclusion. After reading the full-text articles, four were further excluded (Table S1). Two of the studies identified through the international conference abstracts were also excluded (being duplicates of those identified through databases), leaving one study eligible for inclusion. Finally, we included four eligible studies [4,5,6,7] in the systematic review and meta-analysis, comprising a total of 4732 patients (mean age: 69.3 years; 33% women) that were treated with any factor XIa inhibitor of any dose versus 1798 controls (mean age: 69.1 years; 34% women; Figures S1 and S2).

All studies were phase II dose-finding RCTs [4,5,6,7]. Three studies were published in peer-reviewed journals [5,6,7], while the results of the AXIOMATIC-SSP trial [4] were presented during the European Society of Cardiology Congress 2022 [27] and the World Stroke Congress 2022 [28]. PACIFIC-STROKE and AXIOMATIC-SSP included patients within 48 h after acute IS or high-risk TIA, evaluating the efficacy and safety of the factor XIa inhibitors in the secondary stroke prevention setting [4,7]. It should be noted that administration of factor XIa inhibitors followed acute reperfusion therapies in 13% of the patients included in these trials (Figure S3). A history of previous stroke was not mandatory for inclusion in PACIFIC-AF and PACIFIC-AMI (9% and 5.4% of the included patients had a previous history of stroke/TIA, respectively), which predominantly investigated patients in the primary stroke prevention setting [5,6]. More specifically, PACIFIC-AF included patients with atrial fibrillation, and a CHA_2_DS_2_-VASc score of at least 2 if male or at least 3 if female, while PACIFIC-AMI enrolled patients within 5 days of acute myocardial infarction [5,6]. In AXIOMATIC-SSP, PACIFIC-AMI and PACIFIC-STROKE, the interventional arm consisted of factor XIa inhibitor plus standard of care, which consisted of antiplatelet treatment (dual antiplatelet treatment was administered in 92% of the patients; Figure S4), versus placebo plus standard of care [4,6,7], while in the PACIFIC-AF trial, patients in the interventional arm received factor XIa inhibitor alone (in the absence of standard of care) and were compared to those receiving apixaban (as part of standard of care) [5]. The drug milvexian was used in AXIOMATIC-SSP [4], while the rest of the trials tested asundexian [5,6,7]. The characteristics of the included studies are presented in Table 1.

### 3.2. Quality Control of Included Studies

**Figure 2:**
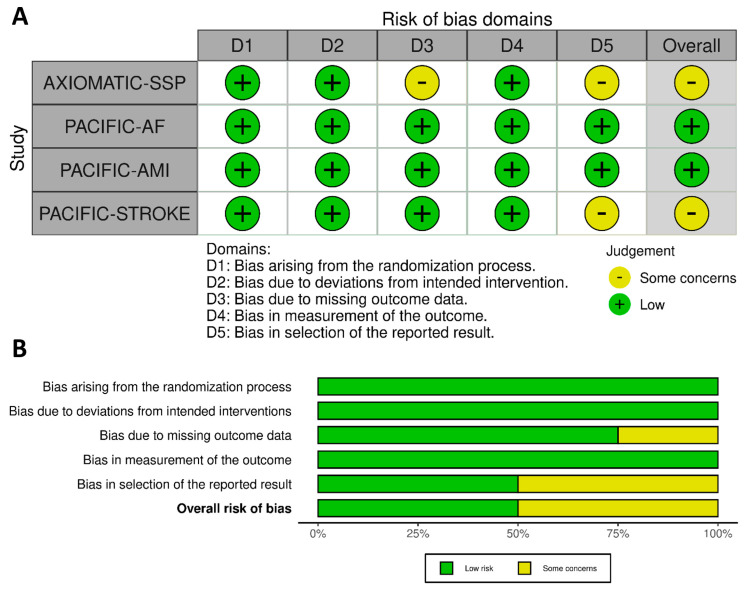
Traffic light plot (**A**) and summary plot (**B**) presenting the quality assessment of included randomized controlled clinical trials using the Cochrane collaboration tool (RoB 2).

The risk of bias of the included studies was assessed using the Cochrane risk-of-bias (RoB 2) tool [19], and is presented in Figure 2. The design and execution of all RCTs were of excellent quality, presenting no bias in the randomization process, or deviations from the intended interventions or the measurements of the outcomes. AXIOMATIC-SSP presented minor concerns due to missing outcome data as a result of study discontinuation and loss to follow-up in less than 5% of the enrolled patients [4]. Finally, minor reporting bias may be attributed to AXIOMATIC-SSP and PACIFIC-STROKE, both of which opted to present post hoc, exploratory outcome data (regarding the composite of symptomatic IS and TIA); however, this was sufficiently underscored in both studies [4,7].

### 3.3. Quantitative Analyses

An overview of the analyses for all primary and secondary efficacy and safety outcomes is presented in Table 2.

### 3.4. Efficacy Outcomes

**Figure 3:**
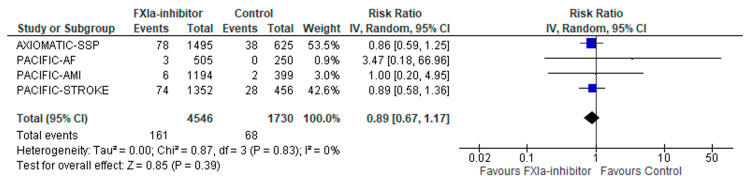
Forest plot presenting the association of factor XIa inhibitors compared to controls with symptomatic ischemic stroke.

There were no differences in the risk of IS among patients receiving factor XIa inhibitors versus controls (RR: 0.89; 95% CI: 0.67–1.17; four studies; I^2^ = 0%; *p* for Cochran Q = 0.83; Figure 3). However, FI was calculated at 10, indicating that the result was “fragile/somewhat robust”. When stratified for different settings, no subgroup differences were shown between primary versus secondary prevention settings (*p* for subgroup differences = 0.57; Figure S5). Similar results were also found after stratification for different factor XIa inhibitors (asundexian versus milvexian; *p* for subgroup differences = 0.80; Figure S6) and different experimental arms (factor XIa inhibitors plus standard of care vs. factor XIa inhibitors alone; *p* for subgroup differences = 0.36; Figure S7).

The composite of symptomatic IS and covert brain infarction was the primary endpoint of AXIOMATIC-SSP and PACIFIC-STROKE studies [4,7], while PACIFIC-AF and PACIFIC-AMI did not report this outcome [5,6]. The analysis of the two studies disclosed a similar risk for symptomatic IS and covert brain infarction combined among patients treated with factor XIa inhibitors plus standard of care versus standard of care alone (RR: 1.01; 95% CI: 0.87–1.18; two studies; I^2^ = 0%; *p* for Cochran Q = 0.89; Figure S8). FI was calculated at 27, indicating that the result was “robust”. Both of the included studies in this analysis enrolled patients in secondary prevention settings, and factor XIa inhibitors were administered on top of standard of care; therefore, subgroup analyses stratified for different settings or different experimental arms were not performed. With regard to different factor XIa inhibitors, there was no difference between asundexian and milvexian (*p* for subgroup differences = 0.50; Figure S9).

The composite of symptomatic IS and TIA was also assessed as an exploratory outcome in the same two studies, AXIOMATIC-SSP and PACIFIC-STROKE [4,7]. Patients receiving factor XIa inhibitors on top of standard of care (i.e., antiplatelet treatment) presented a non-significantly lower risk of composite symptomatic IS and TIA at follow-up compared to patients receiving standard of care alone (RR: 0.78; 95% CI: 0.61–1.01; two studies; I^2^ = 0%; *p* for Cochran Q = 0.94; Figure S10). FI was calculated at 1, indicating that the results were “highly fragile/non-robust”. No difference was evident when analysis was stratified for different factor XIa inhibitors (*p* for subgroup differences = 0.94; Figure S11).

AXIOMATIC-SSP was excluded from the analysis of MACE, since all-cause death was reported as a component of MACE rather than cardiovascular death [4]. Analysis of the remaining three studies found no difference in the occurrence of MACE at follow-up (RR: 1.07; 95% CI: 0.87–1.31; three studies; I^2^ = 0%; *p* for Cochran Q = 0.98; Figure S12). FI was calculated at 19, indicating that the result was “robust”. In all three studies included in this analysis, the administered factor XIa inhibitor was asundexian. There was no subgroup difference regarding the association of factor XIa inhibitors and MACE, when stratified for different settings (*p* for subgroup differences = 0.89; Figure S13) or for different experimental arms (*p* for subgroup differences = 0.91; Figure S14).

### 3.5. Safety Outcomes

**Figure 4:**
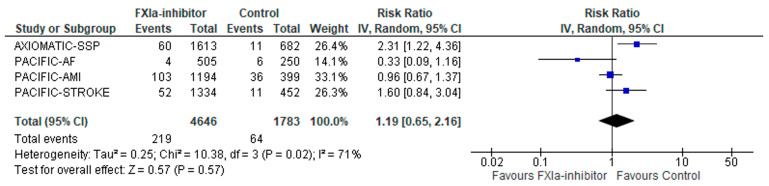
Forest plot presenting the association of factor XIa inhibitors compared to controls with the composite of major bleeding and clinically relevant non-major bleeding.

With regard to the primary safety outcome, the composite of major bleeding or clinically relevant non-major bleeding did not differ among patients receiving factor XIa inhibitor versus controls (RR: 1.19; 95% CI: 0.65–2.16; four studies; I^2^ = 71%; *p* for Cochran Q = 0.02; Figure 4). FI was calculated at 10, indicating that the result was “fragile/somewhat robust”. When stratified for different settings (primary vs. secondary prevention), there was no statistically significant subgroup difference (*p* for subgroup differences = 0.06; Figure S15), although factor XIa inhibitors were associated with a higher risk of major bleeding or clinically relevant non-major bleeding in the secondary stroke prevention setting (RR: 1.92; 95% CI: 1.22–3.02; two studies; I^2^ = 0%; *p* for Cochran Q = 0.43; Figure S15). Similarly, when stratified for different factor XIa inhibitors, there was no statistically significant subgroup difference (*p* for subgroup differences = 0.05; Figure S16), although milvexian was associated with a higher risk of major bleeding or clinically relevant non-major bleeding compared to controls (RR: 2.31; 95% CI: 1.22–4.36; one study; I^2 =^ not applicable; *p* for Cochran Q = not applicable; Figure S16). Conversely, there were significant subgroup differences when the analysis was stratified for different experimental arms (*p* for subgroup differences = 0.03; Figure S17). However, factor XIa inhibitors were not associated with major bleeding or clinically relevant non-major bleeding in any of the subgroups (factor XIa inhibitors plus standard of care: RR: 1.45; 95% CI: 0.83–2.5; three studies; I^2^ = 68%; *p* for Cochran Q = 0.04; factor XIa inhibitors without standard of care: RR: 0.33; 95% CI: 0.09–1.16; one study; I^2^ = not applicable; *p* for Cochran Q = not applicable; Figure S17).

When major bleeding was considered alone, there was again a similar risk between the two arms (RR: 1.19; 95% CI: 0.64–2.22; three studies; I^2^ = 0%; *p* for Cochran Q = 0.43; Figure S18). FI was calculated at 6, indicating that the result was “fragile/somewhat robust”. In the case of major bleeding, no subgroup differences were noted when analysis was stratified based on different settings (*p* for subgroup differences = 0.26; Figure S19) or based on different factor XIa inhibitors (*p* for subgroup differences = 0.25; Figure S20).

Factor XIa inhibitors were not associated with intracranial bleeding (RR: 0.91; 95% CI: 0.26–3.19; three studies; I^2^ = 0%; *p* for Cochran Q = 0.99; Figure S21). FI was calculated at 5, indicating that the result was “fragile/somewhat robust”. This result was not modified after subgroup analysis for either the settings (*p* for subgroup differences = 0.95; Figure S22) or the factor XIa inhibitors used (*p* for subgroup differences = 0.88; Figure S23).

Regarding all-cause mortality, similar risk was found among patients receiving factor XIa inhibitors versus controls (RR: 1.21; 95% CI: 0.77–1.90; four studies; I^2^ = 0%; *p* for Cochran Q = 0.98; Figure S24). FI was calculated at 7, indicating that the result was “fragile/somewhat robust”. No differences emerged in any of the subgroup analyses performed (setting stratification: *p* for subgroup differences = 0.97; Figure S25; factor stratification: *p* for subgroup differences = 0.74; Figure S26; experimental arm stratification: *p* for subgroup differences = 0.81; Figure S27).

During sensitivity analysis, no dose–response relationship was noted for the primary efficacy outcome of IS occurrence (*p* for subgroup differences = 0.89; Figure S28), presenting similar risks when intermediate doses were compared to low doses of factor XIa inhibitor (RR: 0.95; 95% CI: 0.64–1.41; three studies; I^2^ = 0%; *p* for Cochran Q = 0.74) and during comparison of high to low doses of factor XIa inhibitor (RR: 1.00; 95% CI: 0.54–1.84; three studies; I^2^ = 50%; *p* for Cochran Q = 0.14). Likewise, the results of the sensitivity analysis disclosed a similar level of risk for intermediate vs. low doses of factor XIa inhibitor with regard to the composite safety outcome of major bleeding and clinically relevant non-major bleeding (RR: 0.98; 95% CI: 0.70–1.38; three studies; I^2^ = 0%; *p* for Cochran Q = 0.62). Similarly, no difference in risk was identified between high doses and low doses (RR: 1.24; 95% CI: 0.92–1.69; three studies; I^2^ = 0%; *p* for Cochran Q = 0.72). No significant subgroup differences (*p* for subgroup differences = 0.31; Figure S29) were disclosed across the different dosing regimens.

Finally, evaluation for publication bias could not be performed, since only four studies were included in the analysis.

## 4. Discussion

The present meta-analysis shows that treatment with factor XIa inhibitors is not related to lower IS risk compared to controls (RR: 0.89; 95% CI: 0.67–1.17). Likewise, the composite of symptomatic IS and covert infarction on brain MRI (RR: 1.01; 95% CI: 0.87–1.18), the composite of symptomatic IS and TIA (RR: 0.78; 95% CI: 0.61–1.01) and the composite of major adverse cardiovascular events (RR: 1.07; 95% CI: 0.87–1.31) were also similar between the two groups. These results were also confirmed in subgroup analyses stratified for different settings (primary versus secondary stroke prevention), for different factors (asundexian versus milvexian) and different experimental arms (factor XIa inhibitors plus standard of care versus factor XIa inhibitors alone). Furthermore, treatment with factor XIa inhibitors was not associated with any of the assessed safety outcomes. Similar risk was shown for the composite of major bleeding and clinically relevant non-major bleeding (RR: 1.19; 95% CI: 0.65–2.16), major bleeding alone (RR: 1.19; 95% CI: 0.64–2.22), intracranial hemorrhage (RR: 0.91; 95% CI: 0.26–3.19) and all-cause mortality (RR: 1.21; 95% CI: 0.77–1.90).

Based on the above findings, there is a non-significant trend towards IS prevention with factor XIa inhibitors (11% risk reduction without evidence of any heterogeneity). However, it should be underscored that symptomatic IS was not the primary outcome of interest in any of the included phase II RCTs, and thus they were not powered to show statistically significant differences for this outcome. Importantly, all studies were designed as dose-finding phase II RCTs, and all factor XIa inhibitor doses were pooled together for the analyses, at the risk of neutralizing any potential efficacy signals of one particular dose. However, when attempting to stratify the different doses of factor XIa inhibitors administered, no significant dose–response relationship was noted regarding IS occurrence.

The composite of symptomatic IS and covert infarction was the primary efficacy outcome of interest in the two studies that were included in the analysis, AXIOMATIC-SSP and PACIFIC-STROKE [4,7]. Both studies were performed to uncover any signals for harm and assess the dose–response relationship to identify the dose to be used in the subsequent phase III studies. In both studies (and as a result in this meta-analysis, as well), treatment with factor XIa inhibitor plus standard of care was not associated with a reduction in this composite outcome. This seems to have been driven by a complete lack of effect of factor XIa inhibitor on the component of covert infarction, which accounted for the majority of the events of the composite outcome. Importantly, these events were mostly small subcortical infarcts, deemed to be due to cerebral small vessel disease. In Mendelian randomization studies, factor XI levels were not associated with IS attributable to small vessel occlusion [29], partly explaining the failure of treatment with factor XIa inhibitors in small-vessel stroke prevention. The results of the current meta-analysis could inform the design of future RCTs in this setting, questioning the inclusion of covert brain infarction in the primary or secondary efficacy endpoints.

A signal of efficacy, although not statistically significant, was noted while assessing the outcome of the composite symptomatic IS and TIA, where factor XIa inhibitors plus antiplatelet treatment reduced the events by 22% compared to antiplatelet treatment alone. Nevertheless, it should be noted that this outcome was assessed as a post hoc exploratory outcome in both studies included in this analysis [4,7]. Additionally, the reduction in this outcome appears to have been driven by the reduction of TIA rather than symptomatic IS and this may be criticized as a potential limitation. However, despite that the outcome of TIA may not be considered such a hard endpoint, it should be noted that both studies were double-blind and every outcome was also centrally adjudicated by certified stroke physicians, highlighting the objectivity of TIA-reporting in both arms.

Further prespecified subgroup analysis of the PACIFIC-STROKE study also showed that the reduction in the composite symptomatic IS and TIA was augmented among patients with large-artery stroke at baseline and those with any extra- or intracranial atherosclerosis at baseline that received asundexian 50 mg daily [7]. Interestingly, this patient population presents several similarities to that of the COMPASS trial [30], which has already shown the superiority of the combined antithrombotic treatment (aspirin plus rivaroxaban 2.5 mg twice daily) for stroke prevention among patients with clinical atherosclerosis [31]. However, in the COMPASS trial, patients with a recent history of stroke (within the last month) were excluded from enrollment, leaving a gap regarding the optimal prevention management of post-acute stroke patients with atherosclerosis [30]. The optimal secondary prevention in this high-risk stroke subgroup does indeed represent an important unmet need, considering the fact that acute IS or TIA associated with atherosclerosis has a high risk of early stroke recurrence (approximately 6%) within the first 30 days post stroke [32]. To that end, AXIOMATIC-SSP strictly included patients with evidence of atherosclerosis of a perfusing artery to the index stroke, but as a phase II RCT was underpowered to provide conclusive evidence [4]. Furthermore, in AXIOMATIC-SSP, lacunar stroke as the index event was an exclusion criterion [4]. However, due to an amendment of the study protocol that permitted the performance of the baseline brain MRI even after randomization, it is inevitable that patients with lacunar stroke were eventually included, potentially leading to a dilution of the results.

Despite the lack of effect on efficacy outcomes, it is quite encouraging that the safety outcomes were similar between factor XIa inhibitors and controls. With regard to the composite of major bleeding and clinically relevant non-major bleeding, significant subgroup differences emerged between different experimental arms (factor XIa inhibitors plus standard of care versus factor XIa inhibitors alone); however, factor XIa inhibitors were not associated with higher risk in any of the two subgroups. This subgroup difference was anticipated, considering that in AXIOMATIC-SSP, PACIFIC-AMI, and PACIFIC-STROKE factor Xia inhibitor was administered on top of antiplatelet therapy (in 92% dual antiplatelet therapy), while in PACIFIC-AF, factor XIa inhibitor was administered as a single antithrombotic treatment and compared to apixaban. Additionally, although subgroup analysis did not disclose any significant differences regarding different treatment settings, it should be noted that factor XIa inhibitors administered in the secondary stroke prevention setting were associated with a higher risk of major bleeding and clinically relevant non-major bleeding (RR: 1.92; 95% CI: 1.22–3.02). However, this result appears to be driven by the AXIOMATIC-SSP study and, more specifically, the doses of milvexian 50 mg bid or greater. Furthermore, the majority of the events were not major bleeding, but mostly clinically relevant non-major bleeds, usually due to gastrointestinal bleeding requiring some kind of clinical attention. The latter was confirmed through analysis of major bleeding alone, which did not disclose any differences between the treatment arms, either in the overall analysis or in the different subgroup analyses. In addition, intracranial hemorrhage was also similar between the treatment groups. Notably, this result deserves even more attention, considering that 13% of the stroke patients included in AXIOMATIC-SSP and PACIFIC-STROKE had received acute reperfusion therapies before initiating combined antithrombotic treatment.

Our present meta-analysis followed a prespecified protocol and included all available RCTs completed to date, investigating the efficacy and safety of factor XIa inhibitors with regard to stroke prevention (either primary or secondary). Additionally, it provided an estimate of robustness of the presented results, while different potential sources of heterogeneity were addressed. Despite these strengths, several limitations of our study should also be acknowledged. First, our systematic search was able to provide only four RCTs for inclusion, which provided limited data, especially regarding certain outcomes (such as composite of IS and covert infarction or composite of IS and TIA), which should be interpreted with caution. Second, the included population may be considered heterogenous, since studies included patients from both the primary and the secondary prevention settings. However, several subgroup analyses were performed to address potential causes of heterogeneity (different settings, different factor XIa inhibitors, different experimental arms, different doses). Unfortunately, subgroup analysis stratified by different stroke types at baseline (e.g., large-artery stroke vs. other types) was not possible, since the respective data were not available from the included studies. Furthermore, all included studies were phase II dose-finding studies, and all doses of factor XIa inhibitors were pooled together for analysis, potentially resulting in the neutralization of the effects of a particular dose.

Despite those limitations and considering the favorable safety profile and the potential signal of efficacy, especially in populations with pre-existing atherosclerosis, further studies are currently underway. OCEANIC-AF (NCT05643573) is a phase III trial currently recruiting and randomizing patients with atrial fibrillation to receive asundexian versus apixaban. Furthermore, another phase III trial, OCEANIC-STROKE (BAY 2433334), has recently started recruitment, assessing the efficacy and safety of asundexian plus standard of care versus standard of care alone in patients with non-cardioembolic stroke or high-risk TIA. Another phase III RCT, LIBREXIASTROKE (NCT05702034) will also test the safety and efficacy of milvexian plus standard of care versus standard of care alone in the setting of secondary stroke prevention.

## 5. Conclusions

In conclusion, the current meta-analysis provides preliminary evidence regarding the safety of factor XIa inhibitors for primary or secondary stroke prevention. This finding coupled with potential signals of efficacy in reducing IS (and TIA) underscore the importance of embarking on phase III RCTs to provide more robust data regarding the effect of factor XIa inhibition on stroke prevention.

## Figures and Tables

**Table 1:** Characteristics of randomized controlled clinical trials (RCTs) included in the systematic review and meta-analysis.

Study	Period of Enrollment	Population of Interest	Intervention	Controls	Primary Efficacy Outcome	Primary Safety Outcome	Follow-Up	Risk of Bias
AXIOMATIC-SSP [4]	January 2019–December 2021	Patients ≥ 40 years of age with non-cardioembolic, non-lacunar IS or high-risk TIA, within 48 h of the onset of symptoms, and evidence of visible intracranial or extracranial atherosclerotic in a feeding artery	Milvexian (5 different doses) plus DAPT for 21 days followed by SAPT (*n* = 1675)	Placebo plus DAPT for 21 days followed by SAPT (*n* = 691)	Composite of IS plus covert infarction	Major Bleeding	90 days	Some concerns
PACIFIC-AF [5]	January 2020–June 2021	Patients ≥ 45 years of age with atrial fibrillation, a CHA_2_DS_2_-VASc score of at least 2 if male or at least 3 if female, and increased bleeding risk	Asundexian (2 different doses) plus placebo (*n* = 505)	Apixaban plus placebo (*n* = 250)	Composite of CV death, IS, MI or systemic embolism	Composite of major bleeding or clinically relevant non-major bleeding	12 weeks	Low
PACIFIC-AMI [6]	June 2020–July 2021	Patients ≥ 45 years of age, within 5 days of acute MI	Asundexian (3 different doses) plus DAPT (*n* = 1200)	Placebo plus DAPT (*n* = 401)	Composite of CV death, recurrent MI, IS or hemorrhagic stroke, or stent thrombosis.	Composite of major bleeding or clinically relevant non-major bleeding	6–12 months (median: 368 days)	Low
PACIFIC-STROKE [7]	June 2020–July 2021	Patients ≥ 45 years of age, with non-cardioembolic IS, within 48 h of the onset of symptoms	Asundexian (3 different doses) plus APT (*n* = 1352)	Placebo plus APT (*n* = 456)	Composite of IS plus covert infarction	Composite of major bleeding or clinically relevant non-major bleeding	26 weeks	Some concerns

IS: ischemic stroke; TIA: transient ischemic attack; DAPT: dual antiplatelet treatment; SAPT: single antiplatelet treatment; MI: myocardial infarction; CV: cardiovascular; APT: antiplatelet treatment.

**Table 2:** Overview of analyses for primary and secondary outcomes.

Variable	Effect			Fragility		Subgroups Difference * (*p* Value)		
	*n* of Studies	Risk Ratio (95% CI)	I^2^, *p* for Cochran Q	Index	Interpretation	Settings	Factors	Controls
Primary Efficacy Outcome								
Symptomatic IS	4	0.89 (0.67–1.17)	0%; 0.83	10	Fragile/ Somewhat Robust	0.57	0.80	0.36
Secondary Efficacy Outcomes								
Composite of symptomatic IS & covert infarction on brain MRI	2	1.01 (0.87–1.18)	0%; 0.89	27	Robust	NA	0.50	NA
Composite of symptomatic IS & TIA	2	0.78 (0.61–1.01)	0%; 0.94	1	Highly Fragile/ Not Robust	NA	0.94	NA
MACE	3	1.07 (0.87–1.31)	0%; 0.98	19	Robust	0.89	NA	0.91
Primary Safety Outcomes								
Composite of major bleeding & Clinically relevant non-major bleeding	4	1.19 (0.65–2.16)	71%; 0.02	10	Fragile/ Somewhat Robust	0.06	0.05	0.03
Secondary Safety Outcomes								
Major bleeding	3	1.19 (0.64–2.22)	0%; 0.43	6	Fragile/ Somewhat Robust	0.26	0.25	NA
Intracranial hemorrhage	3	0.91 (0.26–3.19)	0%; 0.99	5	Fragile/ Somewhat Robust	0.95	0.88	NA
All-cause mortality	4	1.21 (0.77–1.90)	0%; 0.98	7	Fragile/ Somewhat Robust	0.97	0.74	0.81

* Primary and secondary outcomes of interest were assessed in subgroup analyses stratified by: (i) different settings (primary versus secondary stroke prevention); (ii) different factor XIa inhibitors (asundexian versus milvexian); and (iii) different experimental arms (factor XIa inhibitors plus standard of care vs. factor XIa inhibitors alone). IS: ischemic stroke; TIA: transient ischemic attack; MACE: major adverse cardiovascular outcomes; CI: confidence interval; NA: not applicable.

## Data Availability

All data generated or analyzed during this study are included in this article and its Supplementary Materials.

## Supplementary Materials

The following supporting information can be downloaded at: https://www.mdpi.com/article/10.3390/jcm12175562/s1, Figure S1: Forest plot presenting the pooled mean of age (in years) of the patients enrolled in either arm of the included randomized-controlled clinical trials; Figure S2: Forest plot presenting the pooled proportion of female patients among the total participants enrolled in either arm of the included randomized-controlled clinical trials; Figure S3: Forest plot presenting the pooled proportion of acute ischemic stroke patients receiving acute reperfusion treatments before randomization to factor XIa inhibitor; Figure S4: Forest plot presenting the pooled proportion of patients receiving dual antiplatelet treatment as part of the standard of care on top of factor XIa inhibitors; Figure S5: Forest plot presenting the association of factor XIa inhibitor treatment versus control with symptomatic ischemic stroke occurrence, after stratification for different stroke prevention settings (primary versus secondary); Figure S6: Forest plot presenting the association of factor XIa inhibitor treatment versus control with symptomatic ischemic stroke occurrence, after stratification for different factor XIa inhibitors (asundexian versus milvexian); Figure S7: Forest plot presenting the association of factor XIa inhibitor treatment versus control with symptomatic ischemic stroke occurrence, after stratification for different experimental arms (factor XIa inhibitors plus standard of care vs. factor XIa inhibitors alone); Figure S8: Forest plot presenting the association of factor XIa inhibitor treatment versus control with the composite of symptomatic ischemic stroke occurrence and covert brain infarction; Figure S9: Forest plot presenting the association of factor XIa inhibitor treatment versus control with the composite of symptomatic ischemic stroke occurrence and covert brain infarction, after stratification for different factor XIa inhibitors (asundexian versus milvexian); Figure S10: Forest plot presenting the association of factor XIa inhibitor treatment versus control with the composite of symptomatic ischemic stroke occurrence and transient ischemic attack; Figure S11: Forest plot presenting the association of factor XIa inhibitor treatment versus control with the composite of symptomatic ischemic stroke occurrence and transient ischemic attack, after stratification for different factor XIa inhibitors (asundexian versus milvexian); Figure S12: Forest plot presenting the association of factor XIa inhibitor treatment versus control with the composite of major adverse cardiovascular events; Figure S13: Forest plot presenting the association of factor XIa inhibitor treatment versus control with the composite of major adverse cardiovascular events, after stratification for different stroke prevention settings (primary versus secondary); Figure S14: Forest plot presenting the association of factor XIa inhibitor treatment versus control with the composite of major adverse cardiovascular events, after stratification for different experimental arms (factor XIa inhibitors plus standard of care vs. factor XIa inhibitors alone); Figure S15: Forest plot presenting the association of factor XIa inhibitor treatment versus control with the composite of major bleeding or clinically relevant non-major bleeding, after stratification for different stroke prevention settings (primary versus secondary); Figure S16: Forest plot presenting the association of factor XIa inhibitor treatment versus control with the composite of major bleeding or clinically relevant non-major bleeding, after stratification for different factor XIa inhibitors (asundexian versus milvexian); Figure S17: Forest plot presenting the association of factor XIa inhibitor treatment versus control with the composite of major bleeding or clinically relevant non-major bleeding, after stratification for different experimental arms (factor XIa inhibitors plus standard of care vs. factor XIa inhibitors alone); Figure S18: Forest plot presenting the association of factor XIa inhibitor treatment versus control with major bleeding; Figure S19: Forest plot presenting the association of factor XIa inhibitor treatment versus control with major bleeding, after stratification for different stroke prevention settings (primary versus secondary); Figure S20: Forest plot presenting the association of factor XIa inhibitor treatment versus control with major bleeding, after stratification for different factor XIa inhibitors (asundexian versus milvexian); Figure S21: Forest plot presenting the association of factor XIa inhibitor treatment versus control with intracranial hemorrhage; Figure S22: Forest plot presenting the association of factor XIa inhibitor treatment versus control with intracranial hemorrhage, after stratification for different stroke prevention settings (primary versus secondary); Figure S23: Forest plot presenting the association of factor XIa inhibitor treatment versus control with intracranial hemorrhage, after stratification for different factor XIa inhibitors (asundexian versus milvexian); Figure S24: Forest plot presenting the association of factor XIa inhibitor treatment versus control with all-cause mortality; Figure S25: Forest plot presenting the association of factor XIa inhibitor treatment versus control with all-cause mortality, after stratification for different stroke prevention settings (primary versus secondary); Figure S26: Forest plot presenting the association of factor XIa inhibitor treatment versus control with all-cause mortality, after stratification for different factor XIa inhibitors (asundexian versus milvexian); Figure S27: Forest plot presenting the association of factor XIa inhibitor treatment versus control with all-cause mortality, after stratification for different experimental arms (factor XIa inhibitors plus standard of care vs. factor XIa inhibitors alone); Figure S28: Forest plot presenting the dose effect of factor XIa inhibitors on symptomatic ischemic stroke occurrence, with the low dose as the reference dose, after stratification for intermediate and high doses of factor XIa inhibitor; Figure S29: Forest plot presenting the dose effect of factor XIa inhibitors on the composite of major bleeding or clinically relevant non-major bleeding, with the low dose as the reference dose, after stratification for intermediate and high doses of factor XIa inhibitor; Table S1: Table of excluded studies with reasons for exclusion. References [16,33,34,35] are cited in Supplementary Materials.
