# Supplementary material for: Efficacy and Safety of Oral Factor XIa Inhibitors in Stroke Prevention: A Systematic Review and Meta-Analysis
Source: J Clin Med. 2023 Aug 26;12(17):5562. doi: 10.3390/jcm12175562 (PMC10488897; doi:10.3390/jcm12175562)
Supplement: Supplementary file 1 [file jcm-12-05562-s001.zip › jcm-2484379-supplementary.pdf]

eMethods

Tables:

Table S1. Table of excluded studies with reasons for exclusion.

References used in the Supplement.

Figures:

Figures S1-S29

Methods S1

Complete search algorithm used in MEDLINE and EMBASE search.

("factor XIa inhibitor"[All Fields] OR "factor-XIa"[All Fields] OR "factor-XI"[All Fields] OR "XIa inhibitor"[All Fields] OR "XI inhibitor"[All Fields] OR "XI inhibition"[All Fields] OR "XIa inhibitor"[All Fields] OR "milvexian"[All Fields] OR "asundexian"[All Fields]) AND ("stroke"[All Fields] OR "ischemic stroke"[All Fields] OR "ischaemic stroke"[All Fields])

Complete search algorithm used in SCOPUS search.

(ALL ({stroke} OR {ischemic stroke} OR {ischaemic stroke}) AND ALL ({factor XIa inhibitor} OR {XIa inhibitor} OR {factor-XIa} OR {XIa inhibition} OR {factor XI inhibitor} OR {XI inhibitor} OR {factor-XI} OR {XI inhibition} OR {milvexian} OR {Asundexian}))

Complete search algorithm used in Cochrane Library search.

(All Text "factor XIa inhibitor" OR "XIa inhibitor" OR "factor XI inhibitor" OR "XI inhibitor" OR "XIa inhibition" OR "XI inhibition") AND (All Text “stroke)

Table S1. Table of excluded studies with reasons for exclusion.

Study Name	Reason(s) for Exclusion
Lim et al.[1]	Editorial
Ma et al.[2]	No report on ischemic stroke
Perera et al.[3]	No report on ischemic stroke
Weitz et al.[4]	No report on ischemic stroke

References used in the Supplement.

1. Lim GB. Novel factor XIa inhibitor reduces bleeding compared with apixaban in atrial fibrillation. Nature reviews Cardiology. 2022;19(6):350.
2. Ma T, Dong Y, Huang L, Yang Y, Geng Y, Fei F, et al. SHR2285, the first selectively oral FXIa inhibitor in China: Safety, tolerability, pharmacokinetics and pharmacodynamics combined with aspirin, clopidogrel or ticagrelor. Frontiers in pharmacology. 2022;13:1027627.
3. Perera V, Wang Z, Lubin S, Ueno T, Shiozaki T, Chen W, et al. Safety, pharmacokinetics, and pharmacodynamics of milvexian in healthy Japanese participants. Scientific reports. 2022;12(1):5165.
4. Weitz JI, Strony J, Ageno W, Gailani D, Hylek EM, Lassen MR, et al. Milvexian for the Prevention of Venous Thromboembolism. The New England journal of medicine. 2021;385(23):2161-72.

Figure S1. Forest plot presenting the pooled mean of age (in years) of the patients enrolled in either arm of the included randomized-controlled clinical trials.

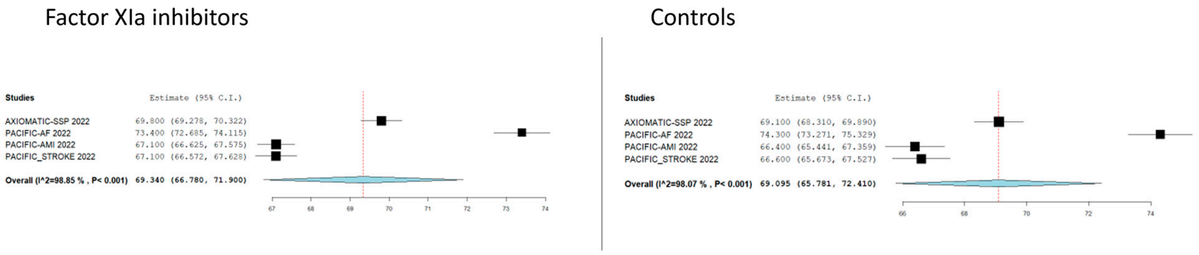

Figure S2. Forest plot presenting the pooled proportion of female patients among the total participants enrolled in either arm of the included randomized-controlled clinical trials.

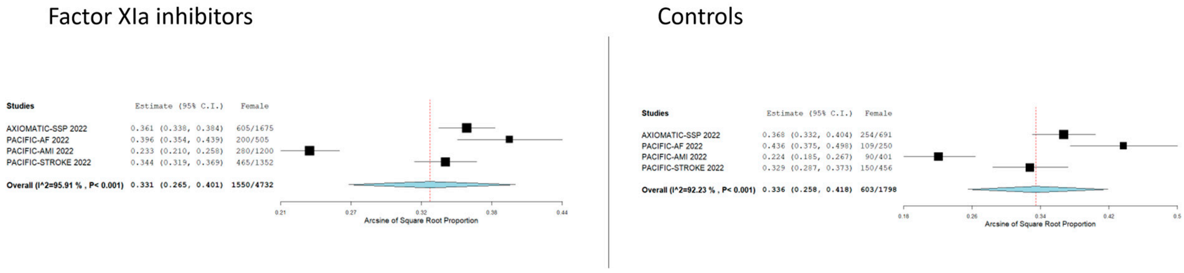

Figure S3. Forest plot presenting the pooled proportion of acute ischemic stroke patients receiving acute reperfusion treatments before randomization to factor XIa inhibitor.

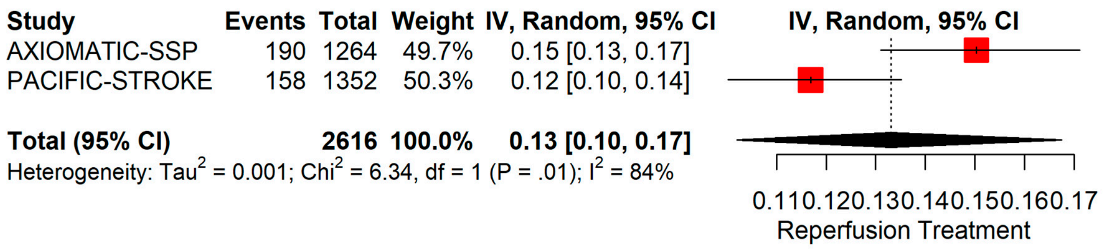

Figure S4. Forest plot presenting the pooled proportion of patients receiving dual antiplatelet treatment as part of the standard of care on top of factor XIa inhibitors.

**Study**	**Events**	**Total**	**Weight**	**IV, Random, 95% CI**
AXIOMATIC-SSP	1675	1675	33.3%	1.00 [1.00, 1.00]
PACIFIC-AMI	1200	1200	33.3%	1.00 [1.00, 1.00]
PACIFIC-STROKE	584	1352	33.3%	0.43 [0.41, 0.46]
**Total (95% CI)**		**4227**	**100.0%**	**0.92 [0.45, 1.00]**

Heterogeneity: $Tau^2 = 0.240$; $Chi^2 = 2680.46$, df = 2 (P = 0); $I^2 = 100\%$

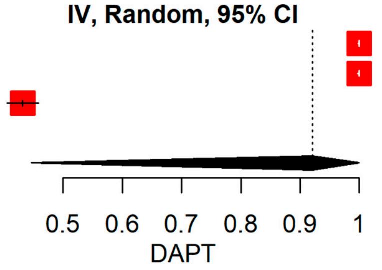

Figure S5: Forest plot presenting the association of factor XIa inhibitor treatment versus control with symptomatic ischemic stroke occurrence, after stratification for different stroke prevention settings (primary versus secondary).

Study or Subgroup	FXIa-inhibitor Events	FXIa-inhibitor Total	Control Events	Control Total	Weight	Risk Ratio IV, Random, 95% CI
**1.2.1 Primary stroke prevention**						
PACIFIC-AF	3	505	0	250	0.9%	3.47 [0.18, 66.96]
PACIFIC-AMI	6	1194	2	399	3.0%	1.00 [0.20, 4.95]
**Subtotal (95% CI)**		**1699**		**649**	**3.8%**	**1.33 [0.33, 5.41]**
Total events	9		2			
Heterogeneity: Tau² = 0.00; Chi² = 0.52, df = 1 (P = 0.47); I² = 0%						
Test for overall effect: Z = 0.39 (P = 0.69)						
**1.2.2 Secondary stroke prevention**						
AXIOMATIC-SSP	78	1495	38	625	53.5%	0.86 [0.59, 1.25]
PACIFIC-STROKE	74	1352	28	456	42.6%	0.89 [0.58, 1.36]
**Subtotal (95% CI)**		**2847**		**1081**	**96.2%**	**0.87 [0.66, 1.16]**
Total events	152		66			
Heterogeneity: Tau² = 0.00; Chi² = 0.02, df = 1 (P = 0.90); I² = 0%						
Test for overall effect: Z = 0.95 (P = 0.34)						
**Total (95% CI)**		**4546**		**1730**	**100.0%**	**0.89 [0.67, 1.17]**
Total events	161		68			
Heterogeneity: Tau² = 0.00; Chi² = 0.87, df = 3 (P = 0.83); I² = 0%						
Test for overall effect: Z = 0.85 (P = 0.39)						
Test for subgroup differences: Chi² = 0.33, df = 1 (P = 0.57), I² = 0%						

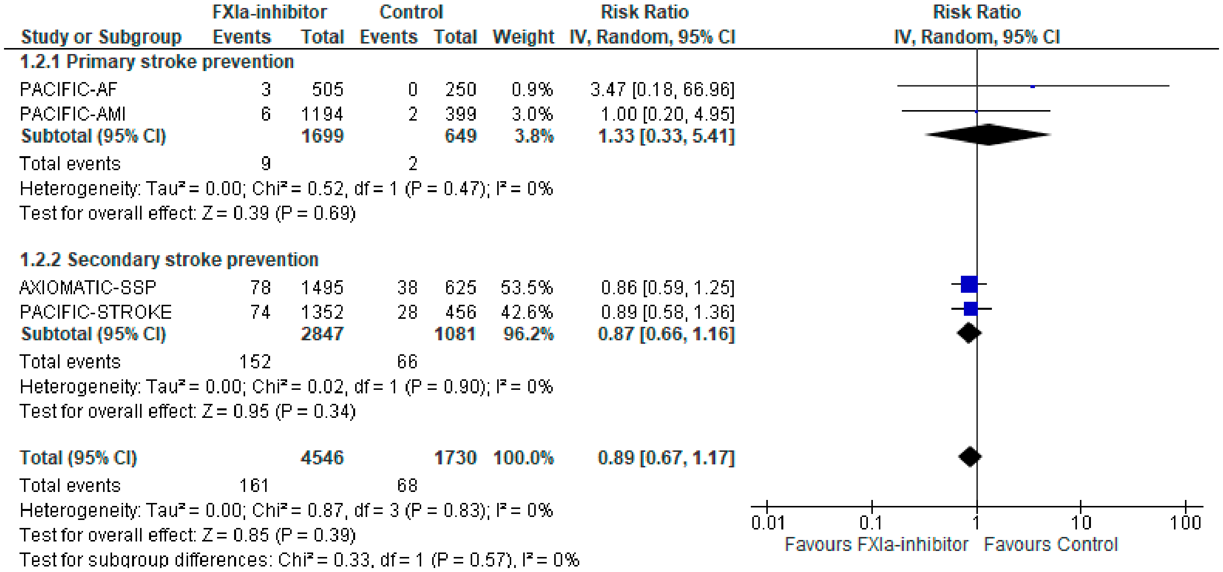

Figure S6: Forest plot presenting the association of factor XIa inhibitor treatment versus control with symptomatic ischemic stroke occurrence, after stratification for different factor XIa inhibitors (asundexian versus milvexian).

Study or Subgroup	FXIa-inhibitor Events	FXIa-inhibitor Total	Control Events	Control Total	Weight	Risk Ratio IV, Random, 95% CI
**1.3.1 Asundexian**						
PACIFIC-AF	3	505	0	250	0.9%	3.47 [0.18, 66.96]
PACIFIC-AMI	6	1194	2	399	3.0%	1.00 [0.20, 4.95]
PACIFIC-STROKE	74	1352	28	456	42.6%	0.89 [0.58, 1.36]
**Subtotal (95% CI)**		**3051**		**1105**	**46.5%**	**0.92 [0.62, 1.38]**
Total events	83		30			
Heterogeneity: Tau² = 0.00; Chi² = 0.81, df = 2 (P = 0.67); I² = 0%						
Test for overall effect: Z = 0.40 (P = 0.69)						
**1.3.2 Milvexian**						
AXIOMATIC-SSP	78	1495	38	625	53.5%	0.86 [0.59, 1.25]
**Subtotal (95% CI)**		**1495**		**625**	**53.5%**	**0.86 [0.59, 1.25]**
Total events	78		38			
Heterogeneity: Not applicable						
Test for overall effect: Z = 0.80 (P = 0.43)						
**Total (95% CI)**		**4546**		**1730**	**100.0%**	**0.89 [0.67, 1.17]**
Total events	161		68			
Heterogeneity: Tau² = 0.00; Chi² = 0.87, df = 3 (P = 0.83); I² = 0%						
Test for overall effect: Z = 0.85 (P = 0.39)						
Test for subgroup differences: Chi² = 0.06, df = 1 (P = 0.80), I² = 0%						

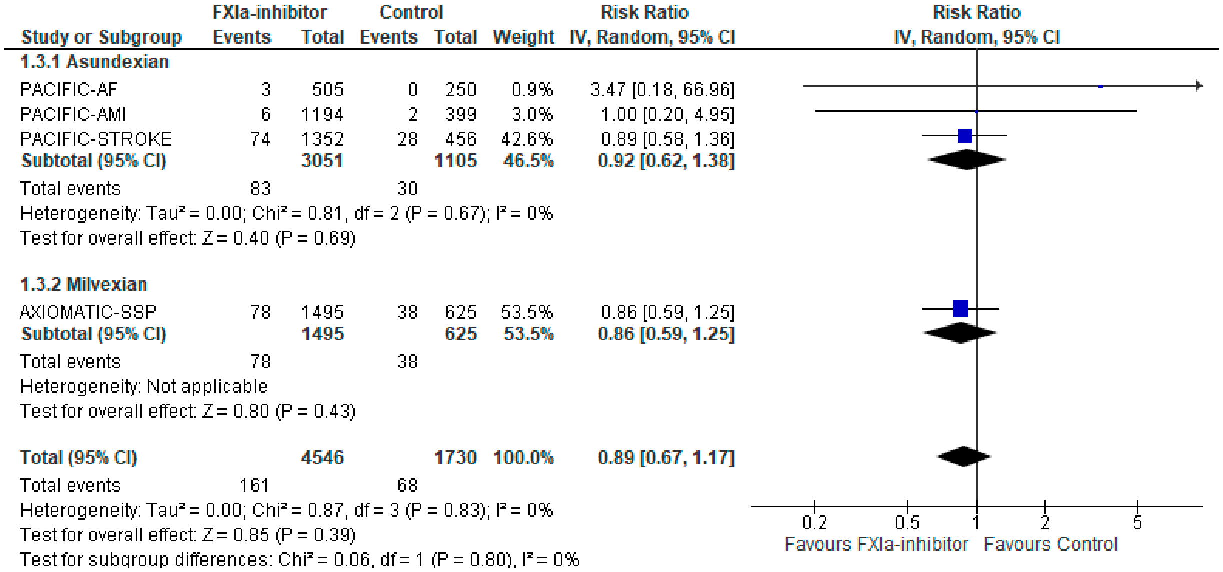

Figure S7: Forest plot presenting the association of factor XIa inhibitor treatment versus control with symptomatic ischemic stroke occurrence, after stratification for different experimental arms (factor XIa inhibitors plus standard of care vs. factor XIa inhibitors alone).

	FXIa-inhibitor		Control			Risk Ratio
**Study or Subgroup**	**Events**	**Total**	**Events**	**Total**	**Weight**	**IV, Random, 95% CI**
**1.4.1 Experimental plus Standard care**						
AXIOMATIC-SSP	78	1495	38	625	53.5%	0.86 [0.59, 1.25]
PACIFIC-AMI	6	1194	2	399	3.0%	1.00 [0.20, 4.95]
PACIFIC-STROKE	74	1352	28	456	42.6%	0.89 [0.58, 1.36]
**Subtotal (95% CI)**		**4041**		**1480**	**99.1%**	**0.88 [0.66, 1.16]**
Total events	158		68			
Heterogeneity: Tau² = 0.00; Chi² = 0.05, df = 2 (P = 0.98); I² = 0%						
Test for overall effect: Z = 0.94 (P = 0.35)						
**1.4.2 Experimental without Standard care**						
PACIFIC-AF	3	505	0	250	0.9%	3.47 [0.18, 66.96]
**Subtotal (95% CI)**		**505**		**250**	**0.9%**	**3.47 [0.18, 66.96]**
Total events	3		0			
Heterogeneity: Not applicable						
Test for overall effect: Z = 0.82 (P = 0.41)						
**Total (95% CI)**		**4546**		**1730**	**100.0%**	**0.89 [0.67, 1.17]**
Total events	161		68			
Heterogeneity: Tau² = 0.00; Chi² = 0.87, df = 3 (P = 0.83); I² = 0%						
Test for overall effect: Z = 0.85 (P = 0.39)						
Test for subgroup differences: Chi² = 0.82, df = 1 (P = 0.36), I² = 0%						

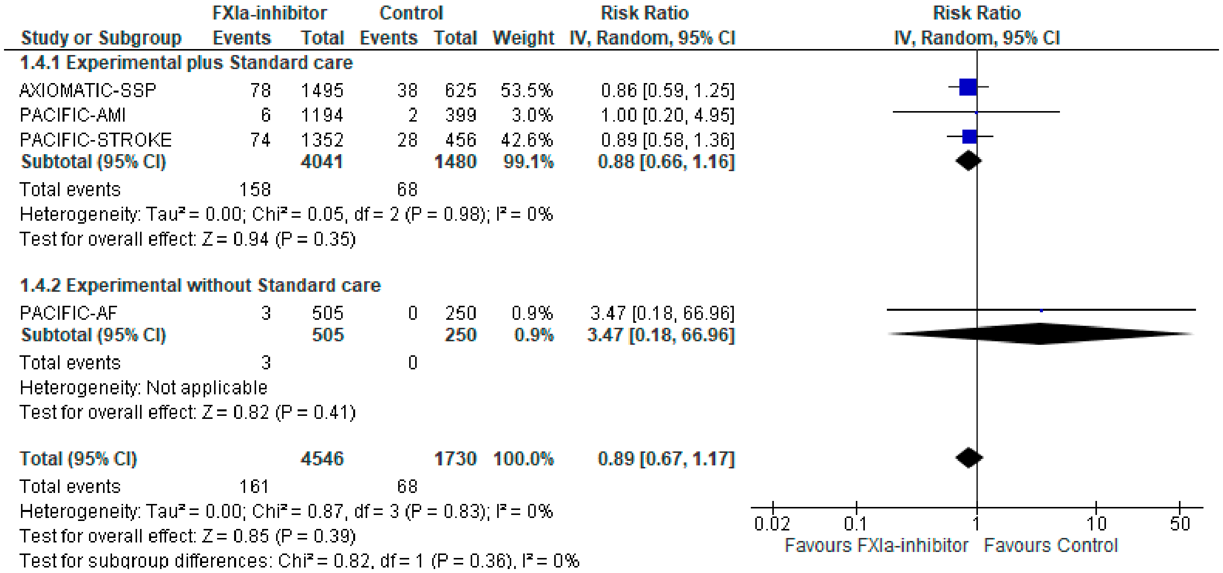

Figure S8: Forest plot presenting the association of factor XIa inhibitor treatment versus control with the composite of symptomatic ischemic stroke occurrence and covert brain infarction.

Study or Subgroup	FXIa-inhibitor Events	FXIa-inhibitor Total	Control Events	Control Total	Weight	Risk Ratio IV, Random, 95% CI
AXIOMATIC-SSP	239	1495	104	625	51.4%	0.96 [0.78, 1.19]
PACIFIC-STROKE	275	1352	87	456	48.6%	1.07 [0.86, 1.32]
**Total (95% CI)**		**2847**		**1081**	**100.0%**	**1.01 [0.87, 1.18]**
Total events	514		191			

Heterogeneity: Tau² = 0.00; Chi² = 0.46, df = 1 (P = 0.50); I² = 0%
Test for overall effect: Z = 0.14 (P = 0.89)

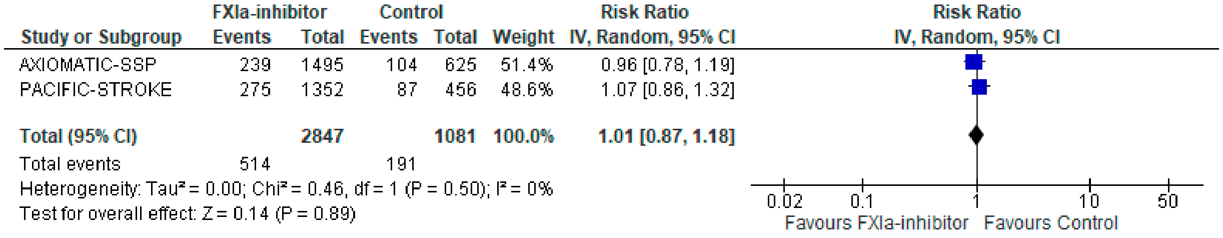

Figure S9: Forest plot presenting the association of factor XIa inhibitor treatment versus control with the composite of symptomatic ischemic stroke occurrence and covert brain infarction, after stratification for different factor XIa inhibitors (asundexian versus milvexian).

	FXIa-inhibitor		Control			Risk Ratio
**Study or Subgroup**	**Events**	**Total**	**Events**	**Total**	**Weight**	**IV, Random, 95% CI**
**1.6.1 Asundexian**						
PACIFIC-STROKE	275	1352	87	456	48.6%	1.07 [0.86, 1.32]
**Subtotal (95% CI)**		**1352**		**456**	**48.6%**	**1.07 [0.86, 1.32]**
Total events	275		87			
Heterogeneity: Not applicable						
Test for overall effect: Z = 0.58 (P = 0.56)						
**1.6.2 Milvexian**						
AXIOMATIC-SSP	239	1495	104	625	51.4%	0.96 [0.78, 1.19]
**Subtotal (95% CI)**		**1495**		**625**	**51.4%**	**0.96 [0.78, 1.19]**
Total events	239		104			
Heterogeneity: Not applicable						
Test for overall effect: Z = 0.37 (P = 0.71)						
**Total (95% CI)**		**2847**		**1081**	**100.0%**	**1.01 [0.87, 1.18]**
Total events	514		191			
Heterogeneity: Tau² = 0.00; Chi² = 0.46, df = 1 (P = 0.50); I² = 0%						
Test for overall effect: Z = 0.14 (P = 0.89)						
Test for subgroup differences: Chi² = 0.46, df = 1 (P = 0.50), I² = 0%						

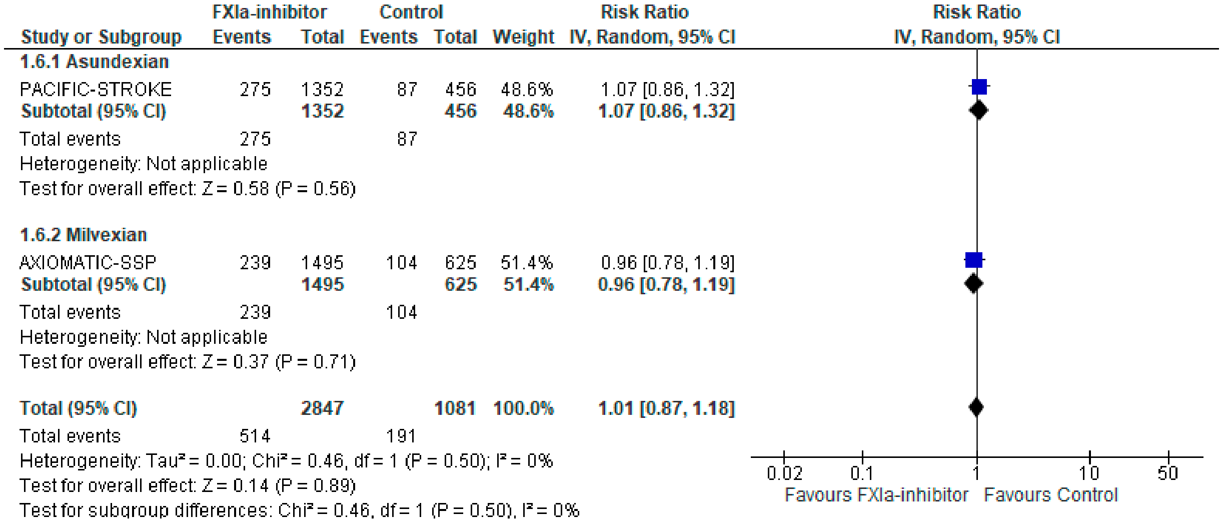

Figure S10: Forest plot presenting the association of factor XIa inhibitor treatment versus control with the composite of symptomatic ischemic stroke occurrence and transient ischemic attack.

	FXIa-inhibitor		Control			Risk Ratio
**Study or Subgroup**	**Events**	**Total**	**Events**	**Total**	**Weight**	**IV, Random, 95% CI**
AXIOMATIC-SSP	83	1495	44	625	51.8%	0.79 [0.55, 1.12]
PACIFIC-STROKE	87	1352	38	456	48.2%	0.77 [0.54, 1.11]
**Total (95% CI)**		**2847**		**1081**	**100.0%**	**0.78 [0.61, 1.01]**
Total events	170		82			

Heterogeneity: Tau² = 0.00; Chi² = 0.01, df = 1 (P = 0.94); I² = 0%

Test for overall effect: Z = 1.91 (P = 0.06)

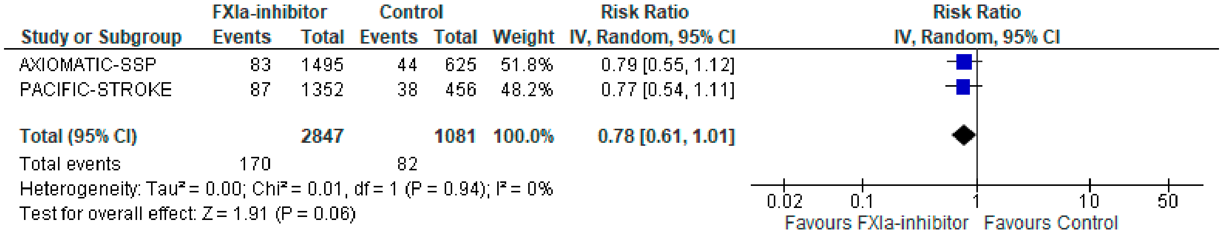

Figure S11: Forest plot presenting the association of factor XIa inhibitor treatment versus control with the composite of symptomatic ischemic stroke occurrence and transient ischemic attack, after stratification for different factor XIa inhibitors (asundexian versus milvexian).

Study or Subgroup	FXIa-inhibitor Events	FXIa-inhibitor Total	Control Events	Control Total	Weight	Risk Ratio IV, Random, 95% CI
**1.8.1 Asundexian**						
PACIFIC-STROKE	87	1352	38	456	48.2%	0.77 [0.54, 1.11]
**Subtotal (95% CI)**		**1352**		**456**	**48.2%**	**0.77 [0.54, 1.11]**
Total events	87		38			
Heterogeneity: Not applicable						
Test for overall effect: Z = 1.38 (P = 0.17)						
**1.8.2 Milvexian**						
AXIOMATIC-SSP	83	1495	44	625	51.8%	0.79 [0.55, 1.12]
**Subtotal (95% CI)**		**1495**		**625**	**51.8%**	**0.79 [0.55, 1.12]**
Total events	83		44			
Heterogeneity: Not applicable						
Test for overall effect: Z = 1.32 (P = 0.19)						
**Total (95% CI)**		**2847**		**1081**	**100.0%**	**0.78 [0.61, 1.01]**
Total events	170		82			
Heterogeneity: Tau² = 0.00; Chi² = 0.01, df = 1 (P = 0.94); I² = 0%						
Test for overall effect: Z = 1.91 (P = 0.06)						
Test for subgroup differences: Chi² = 0.01, df = 1 (P = 0.94), I² = 0%						

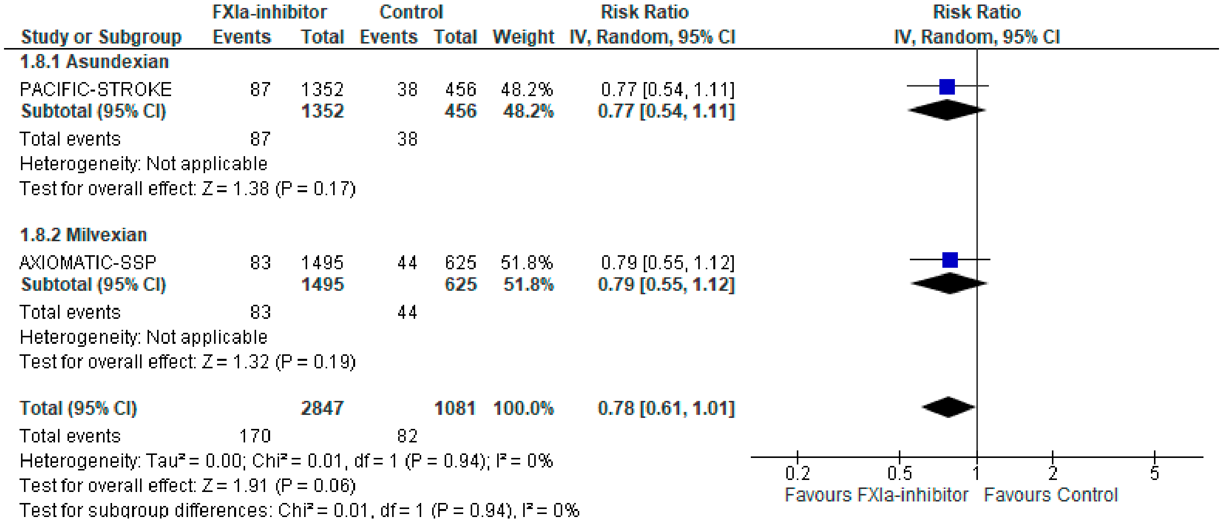

Figure S12: Forest plot presenting the association of factor XIa inhibitor treatment versus control with the composite of major adverse cardiovascular events.

Study or Subgroup	FXIa-inhibitor Events	FXIa-inhibitor Total	Control Events	Control Total	Weight	Risk Ratio IV, Random, 95% CI
PACIFIC-AF	6	505	3	250	2.2%	0.99 [0.25, 3.93]
PACIFIC-AMI	73	1194	22	399	19.3%	1.11 [0.70, 1.76]
PACIFIC-STROKE	248	1352	79	456	78.5%	1.06 [0.84, 1.33]
**Total (95% CI)**		**3051**		**1105**	**100.0%**	**1.07 [0.87, 1.31]**
Total events	327		104			

Heterogeneity: $Tau^2 = 0.00$; $Chi^2 = 0.04$, df = 2 (P = 0.98); $I^2 = 0\%$
Test for overall effect: Z = 0.62 (P = 0.53)

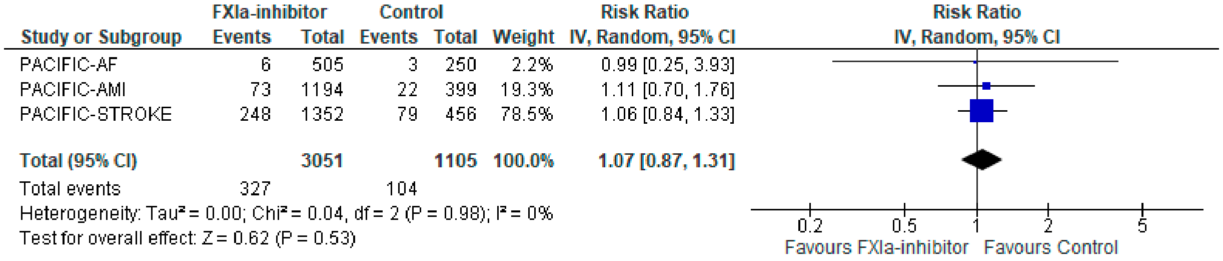

Figure S13: Forest plot presenting the association of factor XIa inhibitor treatment versus control with the composite of major adverse cardiovascular events, after stratification for different stroke prevention settings (primary versus secondary).

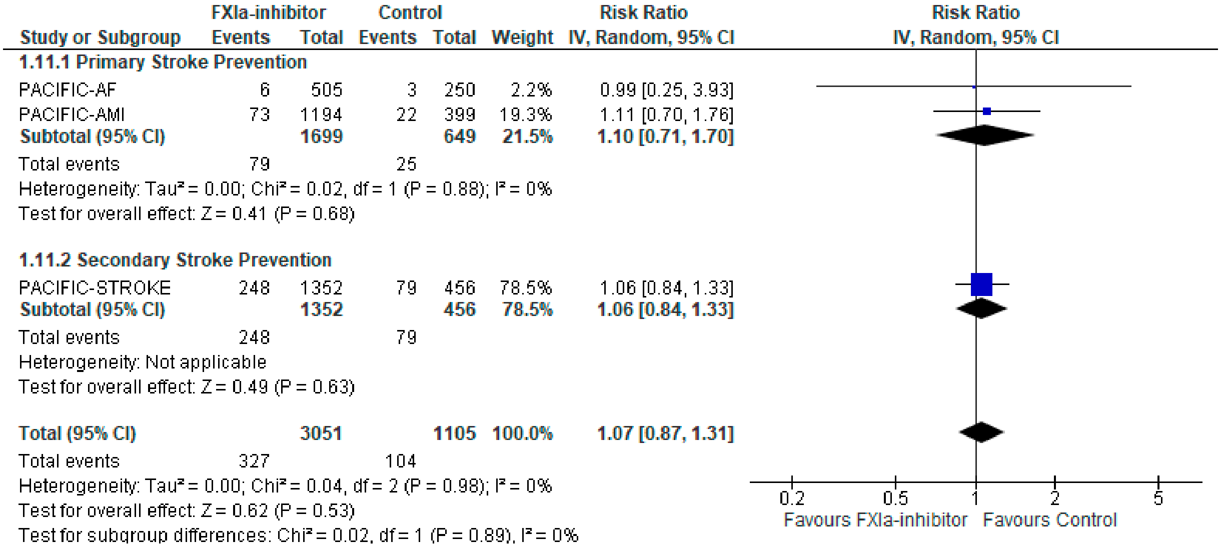

Figure S14: Forest plot presenting the association of factor XIa inhibitor treatment versus control with the composite of major adverse cardiovascular events, after stratification for different experimental arms (factor XIa inhibitors plus standard of care vs. factor XIa inhibitors alone).

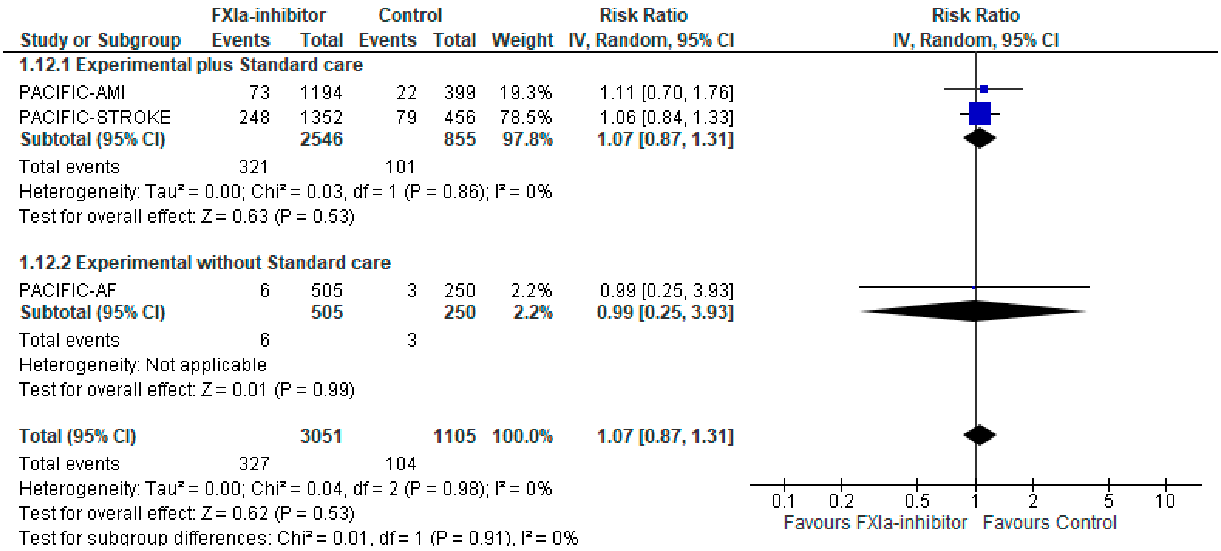

Figure S15: Forest plot presenting the association of factor XIa inhibitor treatment versus control with the composite of major bleeding or clinically relevant non-major bleeding, after stratification for different stroke prevention settings (primary versus secondary).

Study or Subgroup	FXIa-inhibitor Events	FXIa-inhibitor Total	Control Events	Control Total	Weight	Risk Ratio IV, Random, 95% CI
**1.14.1 Primary Stroke Prevention**						
PACIFIC-AF	4	505	6	250	14.1%	0.33 [0.09, 1.16]
PACIFIC-AMI	103	1194	36	399	33.1%	0.96 [0.67, 1.37]
**Subtotal (95% CI)**		**1699**		**649**	**47.3%**	**0.67 [0.25, 1.79]**
Total events	107		42			
Heterogeneity: Tau² = 0.34; Chi² = 2.54, df = 1 (P = 0.11); I² = 61%						
Test for overall effect: Z = 0.80 (P = 0.43)						
**1.14.2 Secondary Stroke Prevention**						
AXIOMATIC-SSP	60	1613	11	682	26.4%	2.31 [1.22, 4.36]
PACIFIC-STROKE	52	1334	11	452	26.3%	1.60 [0.84, 3.04]
**Subtotal (95% CI)**		**2947**		**1134**	**52.7%**	**1.92 [1.22, 3.02]**
Total events	112		22			
Heterogeneity: Tau² = 0.00; Chi² = 0.62, df = 1 (P = 0.43); I² = 0%						
Test for overall effect: Z = 2.84 (P = 0.005)						
**Total (95% CI)**		**4646**		**1783**	**100.0%**	**1.19 [0.65, 2.16]**
Total events	219		64			
Heterogeneity: Tau² = 0.25; Chi² = 10.38, df = 3 (P = 0.02); I² = 71%						
Test for overall effect: Z = 0.57 (P = 0.57)						
Test for subgroup differences: Chi² = 3.65, df = 1 (P = 0.06), I² = 72.6%						

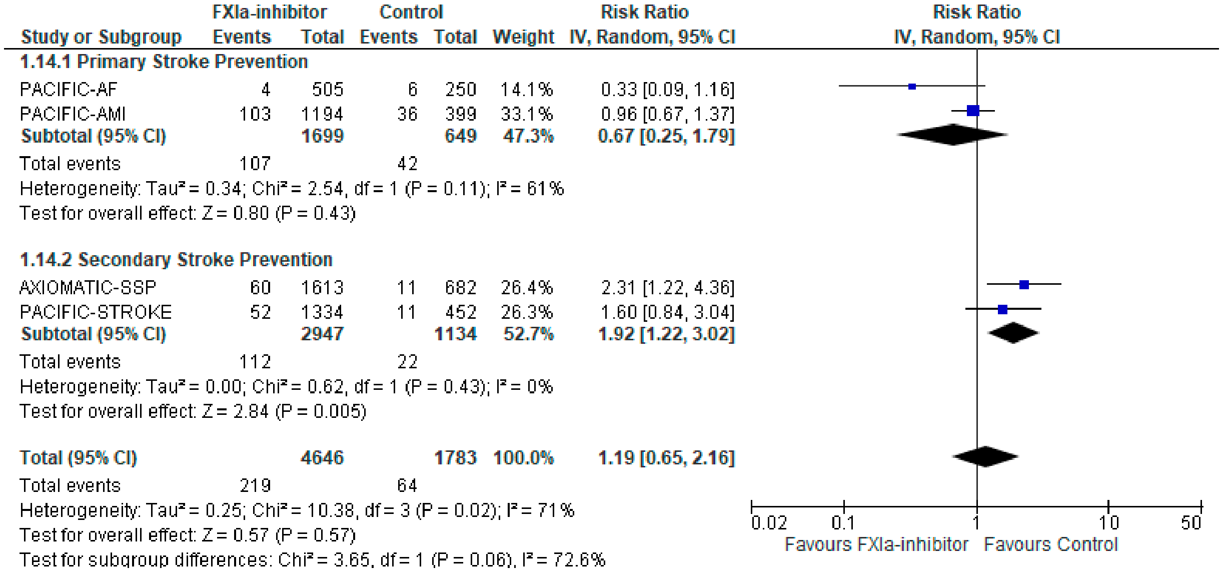

Figure S16: Forest plot presenting the association of factor XIa inhibitor treatment versus control with the composite of major bleeding or clinically relevant non-major bleeding, after stratification for different factor XIa inhibitors (asundexian versus milvexian).

Study or Subgroup	FXIa-inhibitor Events	FXIa-inhibitor Total	Control Events	Control Total	Weight	Risk Ratio IV, Random, 95% CI
**1.15.1 Asundexian**						
PACIFIC-AF	4	505	6	250	14.1%	0.33 [0.09, 1.16]
PACIFIC-AMI	103	1194	36	399	33.1%	0.96 [0.67, 1.37]
PACIFIC-STROKE	52	1334	11	452	26.3%	1.60 [0.84, 3.04]
**Subtotal (95% CI)**		**3033**		**1101**	**73.6%**	**0.96 [0.52, 1.78]**
Total events	159		53			
Heterogeneity: Tau² = 0.17; Chi² = 5.12, df = 2 (P = 0.08); I² = 61%						
Test for overall effect: Z = 0.14 (P = 0.89)						
**1.15.2 Milvexian**						
AXIOMATIC-SSP	60	1613	11	682	26.4%	2.31 [1.22, 4.36]
**Subtotal (95% CI)**		**1613**		**682**	**26.4%**	**2.31 [1.22, 4.36]**
Total events	60		11			
Heterogeneity: Not applicable						
Test for overall effect: Z = 2.57 (P = 0.01)						
**Total (95% CI)**		**4646**		**1783**	**100.0%**	**1.19 [0.65, 2.16]**
Total events	219		64			
Heterogeneity: Tau² = 0.25; Chi² = 10.38, df = 3 (P = 0.02); I² = 71%						
Test for overall effect: Z = 0.57 (P = 0.57)						
Test for subgroup differences: Chi² = 3.77, df = 1 (P = 0.05), I² = 73.5%						

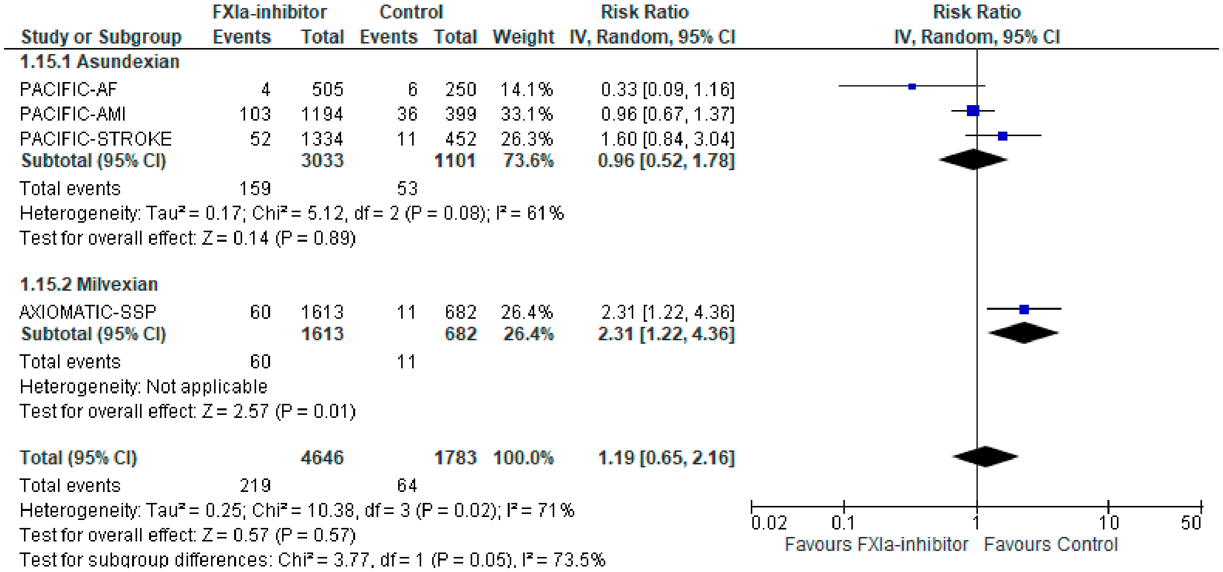

Figure S17: Forest plot presenting the association of factor XIa inhibitor treatment versus control with the composite of major bleeding or clinically relevant non-major bleeding, after stratification for different experimental arms (factor XIa inhibitors plus standard of care vs. factor XIa inhibitors alone).

Study or Subgroup	FXIa-inhibitor Events	FXIa-inhibitor Total	Control Events	Control Total	Weight	Risk Ratio IV, Random, 95% CI
**1.16.1 Experimental plus Standard care**						
AXIOMATIC-SSP	60	1613	11	682	26.4%	2.31 [1.22, 4.36]
PACIFIC-AMI	103	1194	36	399	33.1%	0.96 [0.67, 1.37]
PACIFIC-STROKE	52	1334	11	452	26.3%	1.60 [0.84, 3.04]
**Subtotal (95% CI)**		**4141**		**1533**	**85.9%**	**1.45 [0.83, 2.51]**
Total events	215		58			
Heterogeneity: Tau² = 0.16; Chi² = 6.23, df = 2 (P = 0.04); I² = 68%						
Test for overall effect: Z = 1.32 (P = 0.19)						
**1.16.2 Experimental without Standard care**						
PACIFIC-AF	4	505	6	250	14.1%	0.33 [0.09, 1.16]
**Subtotal (95% CI)**		**505**		**250**	**14.1%**	**0.33 [0.09, 1.16]**
Total events	4		6			
Heterogeneity: Not applicable						
Test for overall effect: Z = 1.73 (P = 0.08)						
**Total (95% CI)**		**4646**		**1783**	**100.0%**	**1.19 [0.65, 2.16]**
Total events	219		64			
Heterogeneity: Tau² = 0.25; Chi² = 10.38, df = 3 (P = 0.02); I² = 71%						
Test for overall effect: Z = 0.57 (P = 0.57)						
Test for subgroup differences: Chi² = 4.46, df = 1 (P = 0.03), I² = 77.6%						

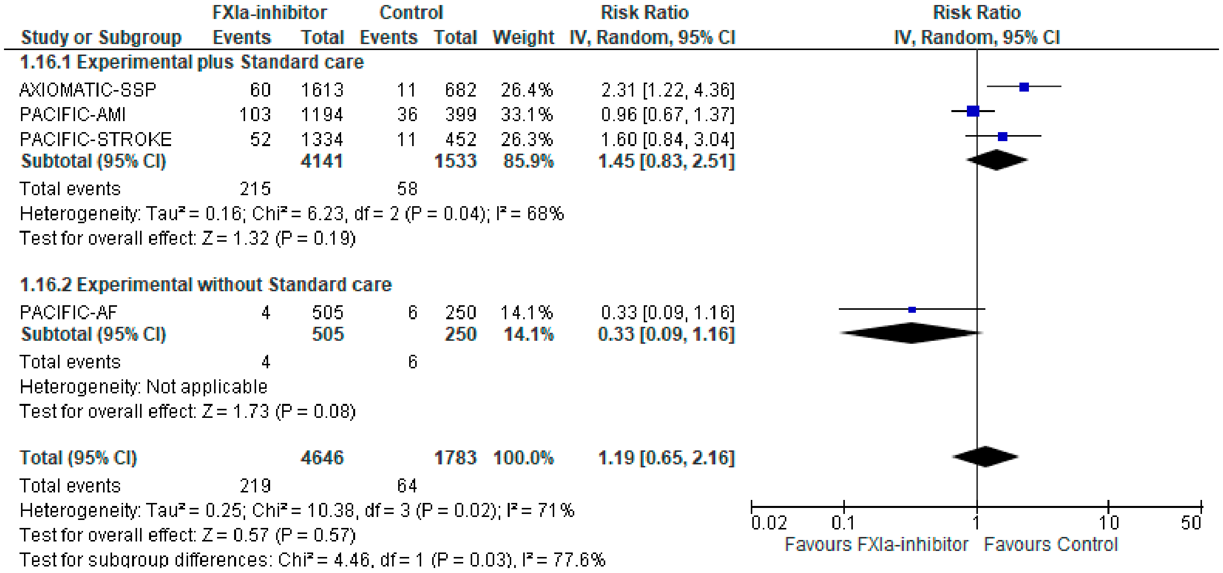

Figure S18: Forest plot presenting the association of factor XIa inhibitor treatment versus control with major bleeding.

	FXIa-inhibitor		Control			Risk Ratio
Study or Subgroup	Events	Total	Events	Total	Weight	IV, Random, 95% CI
AXIOMATIC-SSP	19	1625	4	682	33.5%	1.99 [0.68, 5.84]
PACIFIC-AF	0	505	0	250		Not estimable
PACIFIC-AMI	11	1194	5	399	35.0%	0.74 [0.26, 2.10]
PACIFIC-STROKE	14	1334	4	452	31.6%	1.19 [0.39, 3.58]
**Total (95% CI)**		**4658**		**1783**	**100.0%**	**1.19 [0.64, 2.22]**
Total events	44		13			

Heterogeneity: $Tau^2 = 0.00$; $Chi^2 = 1.69$, df = 2 (P = 0.43); $I^2 = 0\%$
Test for overall effect: Z = 0.56 (P = 0.58)

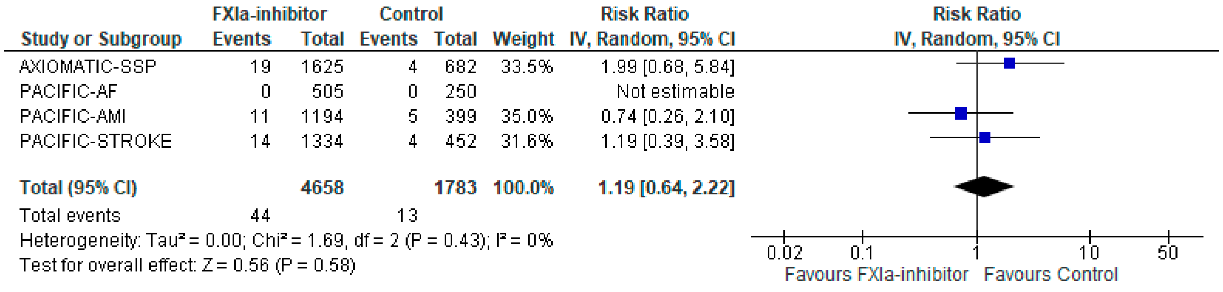

Figure S19: Forest plot presenting the association of factor XIa inhibitor treatment versus control with major bleeding, after stratification for different stroke prevention settings (primary versus secondary).

Study or Subgroup	FXIa-inhibitor Events	FXIa-inhibitor Total	Control Events	Control Total	Weight	Risk Ratio IV, Random, 95% CI
**1.18.1 Primary Stroke Prevention**						
PACIFIC-AMI	11	1194	5	399	35.0%	0.74 [0.26, 2.10]
**Subtotal (95% CI)**		**1194**		**399**	**35.0%**	**0.74 [0.26, 2.10]**
Total events	11		5			
Heterogeneity: Not applicable						
Test for overall effect: Z = 0.57 (P = 0.57)						
**1.18.2 Secondary Stroke Prevention**						
AXIOMATIC-SSP	19	1625	4	682	33.5%	1.99 [0.68, 5.84]
PACIFIC-STROKE	14	1334	4	452	31.6%	1.19 [0.39, 3.58]
**Subtotal (95% CI)**		**2959**		**1134**	**65.0%**	**1.55 [0.72, 3.35]**
Total events	33		8			
Heterogeneity: Tau² = 0.00; Chi² = 0.44, df = 1 (P = 0.51); I² = 0%						
Test for overall effect: Z = 1.11 (P = 0.27)						
**Total (95% CI)**		**4153**		**1533**	**100.0%**	**1.19 [0.64, 2.22]**
Total events	44		13			
Heterogeneity: Tau² = 0.00; Chi² = 1.69, df = 2 (P = 0.43); I² = 0%						
Test for overall effect: Z = 0.56 (P = 0.58)						
Test for subgroup differences: Chi² = 1.26, df = 1 (P = 0.26), I² = 20.4%						

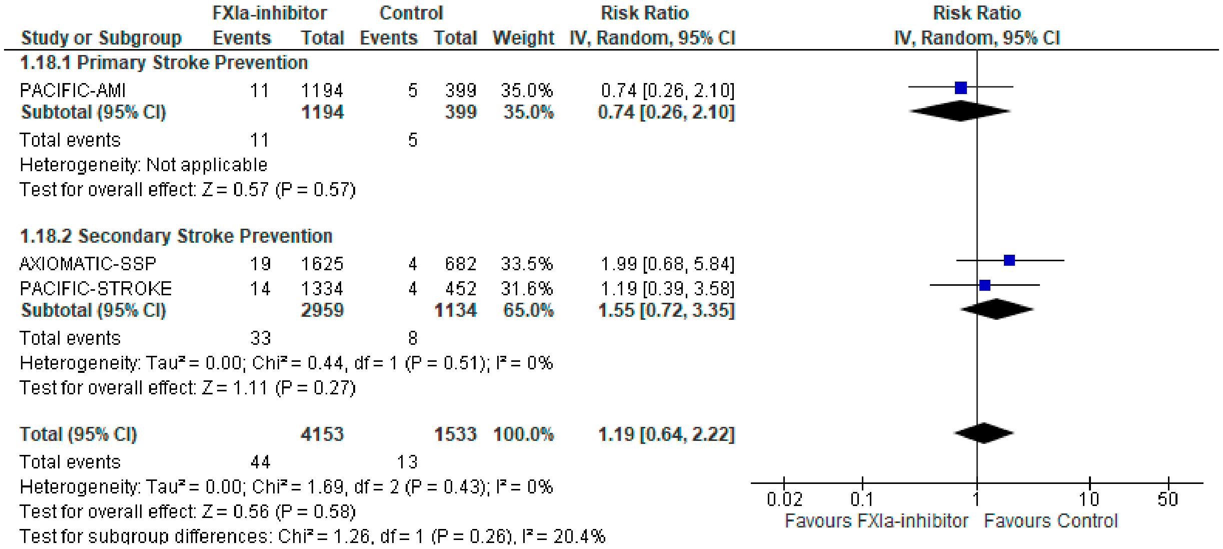

Figure S20: Forest plot presenting the association of factor XIa inhibitor treatment versus control with major bleeding, after stratification for different factor XIa inhibitors (asundexian versus milvexian).

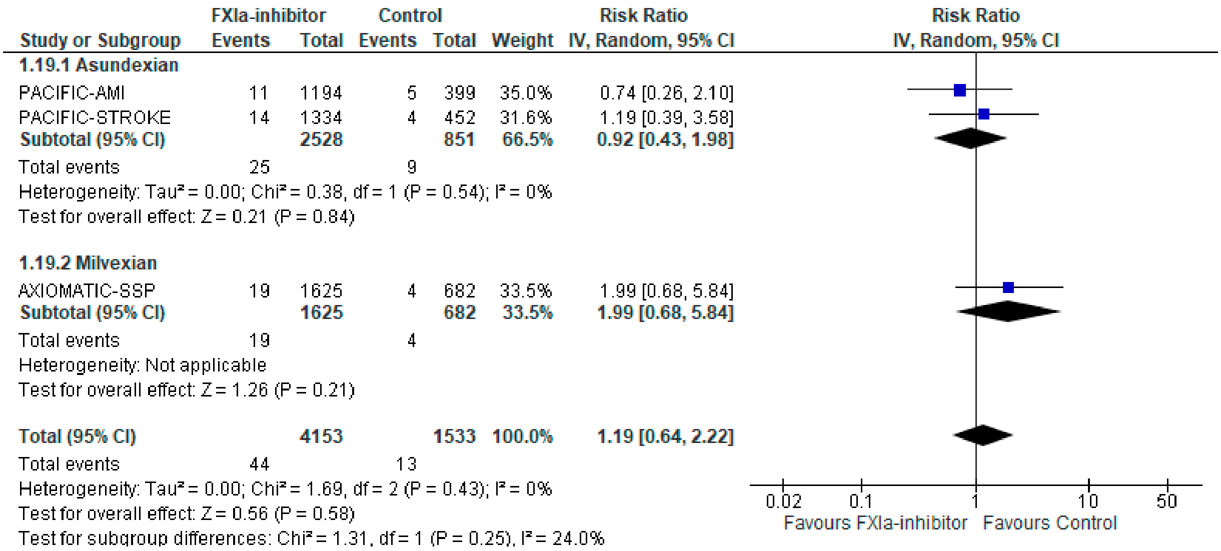

Figure S21: Forest plot presenting the association of factor XIa inhibitor treatment versus control with intracranial hemorrhage.

	FXIa-inhibitor		Control			Risk Ratio
**Study or Subgroup**	**Events**	**Total**	**Events**	**Total**	**Weight**	**IV, Random, 95% CI**
AXIOMATIC-SSP	4	1625	2	682	54.3%	0.84 [0.15, 4.57]
PACIFIC-AMI	1	1194	0	399	15.2%	1.00 [0.04, 24.60]
PACIFIC-STROKE	3	1334	1	452	30.5%	1.02 [0.11, 9.75]
**Total (95% CI)**		**4153**		**1533**	**100.0%**	**0.91 [0.26, 3.19]**
Total events	8		3			

Heterogeneity: $Tau^2 = 0.00$; $Chi^2 = 0.02$, $df = 2$ ($P = 0.99$); $I^2 = 0\%$
Test for overall effect: $Z = 0.14$ ($P = 0.89$)

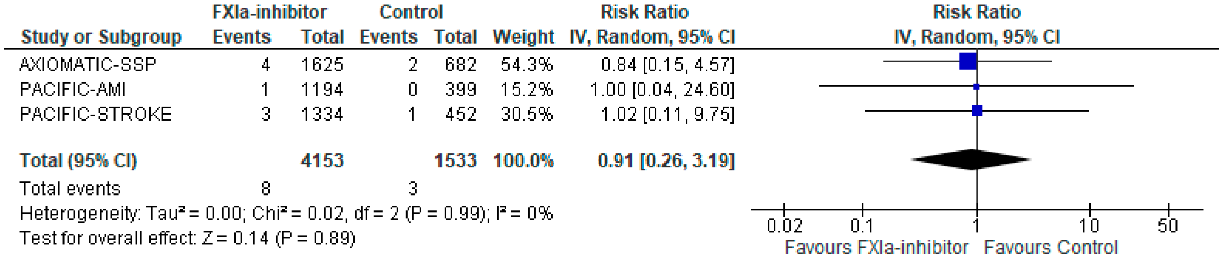

Figure S22: Forest plot presenting the association of factor XIa inhibitor treatment versus control with intracranial hemorrhage, after stratification for different stroke prevention settings (primary versus secondary).

Study or Subgroup	FXIa-inhibitor Events	FXIa-inhibitor Total	Control Events	Control Total	Weight	Risk Ratio IV, Random, 95% CI
**1.21.1 Primary Stroke Prevention**						
PACIFIC-AMI	1	1194	0	399	15.2%	1.00 [0.04, 24.60]
**Subtotal (95% CI)**		**1194**		**399**	**15.2%**	**1.00 [0.04, 24.60]**
Total events	1		0			
Heterogeneity: Not applicable						
Test for overall effect: Z = 0.00 (P = 1.00)						
**1.21.2 Secondary Stroke Prevention**						
AXIOMATIC-SSP	4	1625	2	682	54.3%	0.84 [0.15, 4.57]
PACIFIC-STROKE	3	1334	1	452	30.5%	1.02 [0.11, 9.75]
**Subtotal (95% CI)**		**2959**		**1134**	**84.8%**	**0.90 [0.23, 3.49]**
Total events	7		3			
Heterogeneity: Tau² = 0.00; Chi² = 0.02, df = 1 (P = 0.89); I² = 0%						
Test for overall effect: Z = 0.15 (P = 0.88)						
**Total (95% CI)**		**4153**		**1533**	**100.0%**	**0.91 [0.26, 3.19]**
Total events	8		3			
Heterogeneity: Tau² = 0.00; Chi² = 0.02, df = 2 (P = 0.99); I² = 0%						
Test for overall effect: Z = 0.14 (P = 0.89)						
Test for subgroup differences: Chi² = 0.00, df = 1 (P = 0.95), I² = 0%						

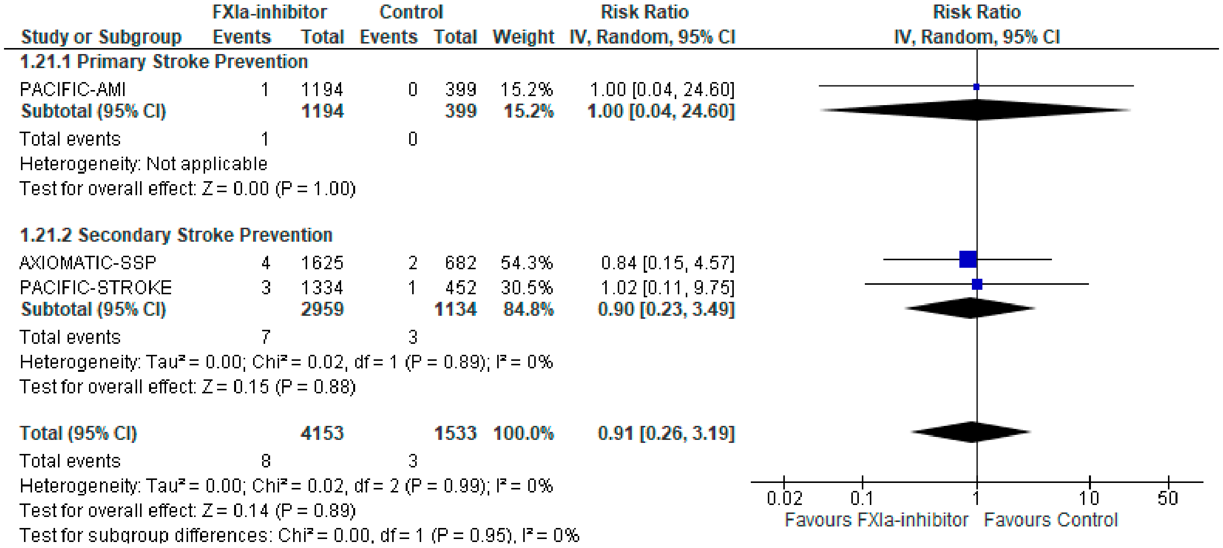

Figure S23: Forest plot presenting the association of factor XIa inhibitor treatment versus control with intracranial hemorrhage, after stratification for different factor XIa inhibitors (asundexian versus milvexian).

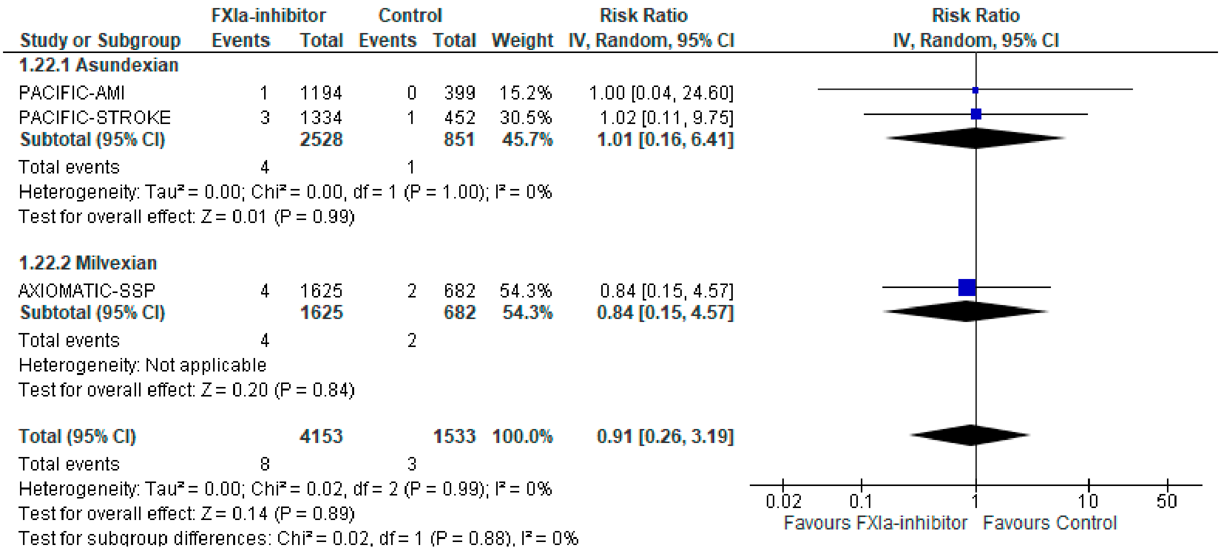

Figure S24: Forest plot presenting the association of factor XIa inhibitor treatment versus control with all-cause mortality.

	FXIa-inhibitor		Control			Risk Ratio
**Study or Subgroup**	**Events**	**Total**	**Events**	**Total**	**Weight**	**IV, Random, 95% CI**
AXIOMATIC-SSP	17	1675	5	691	20.8%	1.40 [0.52, 3.79]
PACIFIC-AF	4	503	2	250	7.2%	0.99 [0.18, 5.39]
PACIFIC-AMI	27	1194	7	399	30.2%	1.29 [0.57, 2.94]
PACIFIC-STROKE	33	1352	10	456	41.9%	1.11 [0.55, 2.24]
**Total (95% CI)**		**4724**		**1796**	**100.0%**	**1.21 [0.77, 1.90]**
Total events	81		24			

Heterogeneity: $Tau^2 = 0.00$; $Chi^2 = 0.21$, df = 3 (P = 0.98); $I^2 = 0\%$

Test for overall effect: Z = 0.83 (P = 0.41)

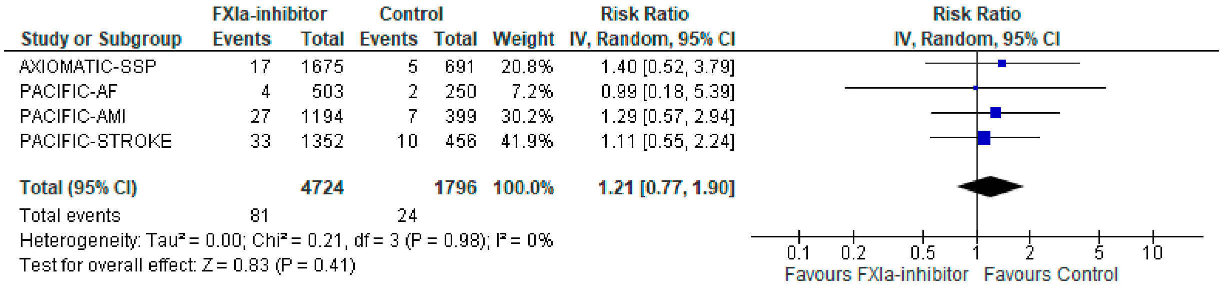

Figure S25: Forest plot presenting the association of factor XIa inhibitor treatment versus control with all-cause mortality, after stratification for different stroke prevention settings (primary versus secondary).

Study or Subgroup	FXIa-inhibitor Events	FXIa-inhibitor Total	Control Events	Control Total	Weight	Risk Ratio IV, Random, 95% CI
**1.24.1 Primary Stroke Prevention**						
PACIFIC-AF	4	503	2	250	7.2%	0.99 [0.18, 5.39]
PACIFIC-AMI	27	1194	7	399	30.2%	1.29 [0.57, 2.94]
**Subtotal (95% CI)**		**1697**		**649**	**37.4%**	**1.23 [0.58, 2.57]**
Total events	31		9			
Heterogeneity: Tau² = 0.00; Chi² = 0.07, df = 1 (P = 0.79); I² = 0%						
Test for overall effect: Z = 0.54 (P = 0.59)						
**1.24.2 Secondary Stroke Prevention**						
AXIOMATIC-SSP	17	1675	5	691	20.8%	1.40 [0.52, 3.79]
PACIFIC-STROKE	33	1352	10	456	41.9%	1.11 [0.55, 2.24]
**Subtotal (95% CI)**		**3027**		**1147**	**62.6%**	**1.20 [0.68, 2.13]**
Total events	50		15			
Heterogeneity: Tau² = 0.00; Chi² = 0.14, df = 1 (P = 0.71); I² = 0%						
Test for overall effect: Z = 0.63 (P = 0.53)						
**Total (95% CI)**		**4724**		**1796**	**100.0%**	**1.21 [0.77, 1.90]**
Total events	81		24			
Heterogeneity: Tau² = 0.00; Chi² = 0.21, df = 3 (P = 0.98); I² = 0%						
Test for overall effect: Z = 0.83 (P = 0.41)						
Test for subgroup differences: Chi² = 0.00, df = 1 (P = 0.97), I² = 0%						

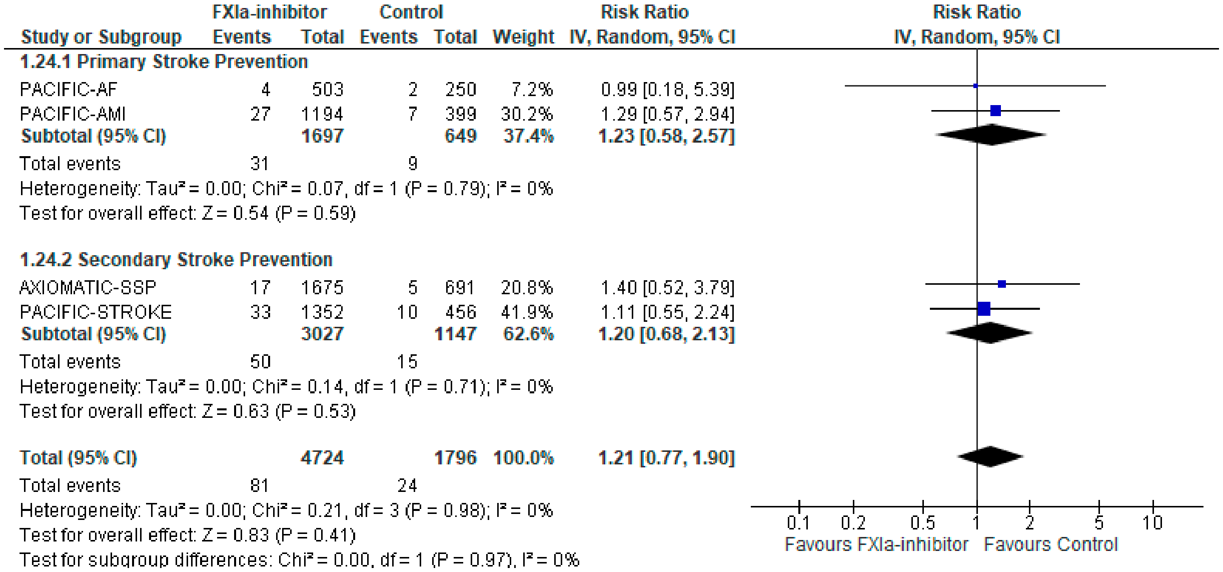

Figure S26: Forest plot presenting the association of factor XIa inhibitor treatment versus control with all-cause mortality, after stratification for different factor XIa inhibitors (asundexian versus milvexian).

Study or Subgroup	FXIa-inhibitor Events	FXIa-inhibitor Total	Control Events	Control Total	Weight	Risk Ratio IV, Random, 95% CI
**1.25.1 Asundexian**						
PACIFIC-AF	4	503	2	250	7.2%	0.99 [0.18, 5.39]
PACIFIC-AMI	27	1194	7	399	30.2%	1.29 [0.57, 2.94]
PACIFIC-STROKE	33	1352	10	456	41.9%	1.11 [0.55, 2.24]
**Subtotal (95% CI)**		**3049**		**1105**	**79.2%**	**1.17 [0.70, 1.94]**
Total events	64		19			
Heterogeneity: Tau² = 0.00; Chi² = 0.11, df = 2 (P = 0.95); I² = 0%						
Test for overall effect: Z = 0.59 (P = 0.56)						
**1.25.2 Milvexian**						
AXIOMATIC-SSP	17	1675	5	691	20.8%	1.40 [0.52, 3.79]
**Subtotal (95% CI)**		**1675**		**691**	**20.8%**	**1.40 [0.52, 3.79]**
Total events	17		5			
Heterogeneity: Not applicable						
Test for overall effect: Z = 0.67 (P = 0.50)						
**Total (95% CI)**		**4724**		**1796**	**100.0%**	**1.21 [0.77, 1.90]**
Total events	81		24			
Heterogeneity: Tau² = 0.00; Chi² = 0.21, df = 3 (P = 0.98); I² = 0%						
Test for overall effect: Z = 0.83 (P = 0.41)						
Test for subgroup differences: Chi² = 0.11, df = 1 (P = 0.74), I² = 0%						

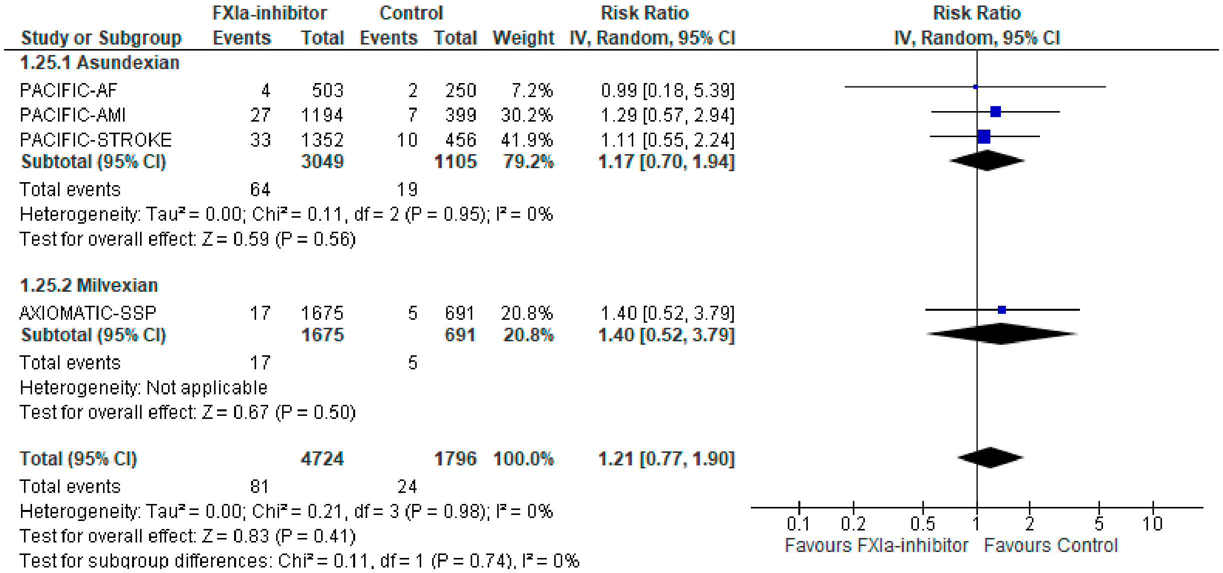

Figure S27: Forest plot presenting the association of factor XIa inhibitor treatment versus control with all-cause mortality, after stratification for different experimental arms (factor XIa inhibitors plus standard of care vs. factor XIa inhibitors alone).

Study or Subgroup	FXIa-inhibitor Events	FXIa-inhibitor Total	Control Events	Control Total	Weight	Risk Ratio IV, Random, 95% CI
**1.26.1 Experimental plus Standard care**						
AXIOMATIC-SSP	17	1675	5	691	20.8%	1.40 [0.52, 3.79]
PACIFIC-AMI	27	1194	7	399	30.2%	1.29 [0.57, 2.94]
PACIFIC-STROKE	33	1352	10	456	41.9%	1.11 [0.55, 2.24]
**Subtotal (95% CI)**		**4221**		**1546**	**92.8%**	**1.23 [0.77, 1.97]**
Total events	77		22			
Heterogeneity: Tau² = 0.00; Chi² = 0.16, df = 2 (P = 0.92); I² = 0%						
Test for overall effect: Z = 0.86 (P = 0.39)						
**1.26.2 Experimental without Standard care**						
PACIFIC-AF	4	503	2	250	7.2%	0.99 [0.18, 5.39]
**Subtotal (95% CI)**		**503**		**250**	**7.2%**	**0.99 [0.18, 5.39]**
Total events	4		2			
Heterogeneity: Not applicable						
Test for overall effect: Z = 0.01 (P = 0.99)						
**Total (95% CI)**		**4724**		**1796**	**100.0%**	**1.21 [0.77, 1.90]**
Total events	81		24			
Heterogeneity: Tau² = 0.00; Chi² = 0.21, df = 3 (P = 0.98); I² = 0%						
Test for overall effect: Z = 0.83 (P = 0.41)						
Test for subgroup differences: Chi² = 0.06, df = 1 (P = 0.81), I² = 0%						

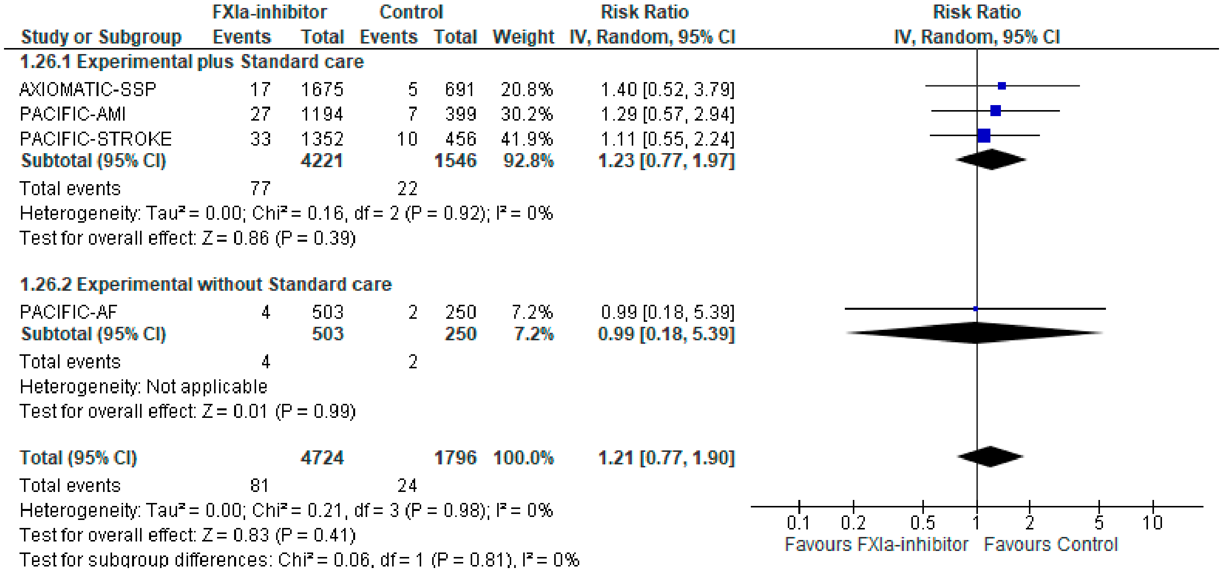

Figure S28: Forest plot presenting the dose effect of factor XIa inhibitors on symptomatic ischemic stroke occurrence, with the low dose as the reference dose, after stratification for intermediate and high doses of factor XIa inhibitor.

	Intermediate Dose		Low Dose			Risk Ratio
**Study or Subgroup**	**Events**	**Total**	**Events**	**Total**	**Weight**	**IV, Random, 95% CI**
AXIOMATIC-SSP	13	328	27	646	37.7%	0.95 [0.50, 1.81]
PACIFIC-AMI	2	397	4	395	5.5%	0.50 [0.09, 2.70]
PACIFIC-STROKE	26	450	26	455	56.8%	1.01 [0.60, 1.71]
**Total (95% CI)**		**1175**		**1496**	**100.0%**	**0.95 [0.64, 1.41]**
Total events	41		57			

Heterogeneity: $Tau^2 = 0.00$; $Chi^2 = 0.62$, df = 2 (P = 0.74); $I^2 = 0\%$
Test for overall effect: Z = 0.26 (P = 0.80)

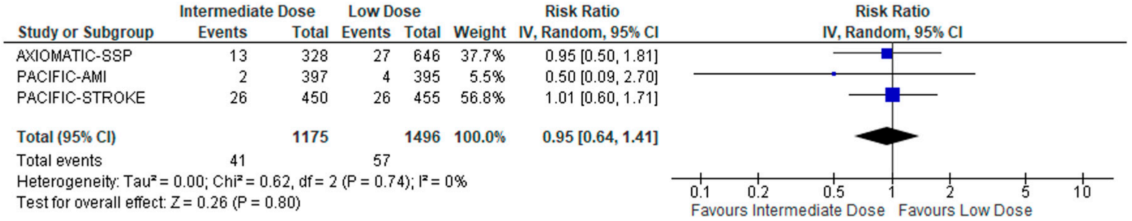

	High Dose		Low Dose			Risk Ratio
**Study or Subgroup**	**Events**	**Total**	**Events**	**Total**	**Weight**	**IV, Random, 95% CI**
AXIOMATIC-SSP	38	661	27	646	50.2%	1.38 [0.85, 2.23]
PACIFIC-AMI	0	402	4	395	4.1%	0.11 [0.01, 2.02]
PACIFIC-STROKE	22	447	26	455	45.7%	0.86 [0.50, 1.50]
**Total (95% CI)**		**1510**		**1496**	**100.0%**	**1.00 [0.54, 1.84]**
Total events	60		57			

Heterogeneity: $Tau^2 = 0.13$; $Chi^2 = 3.98$, df = 2 (P = 0.14); $I^2 = 50\%$
Test for overall effect: Z = 0.00 (P = 1.00)

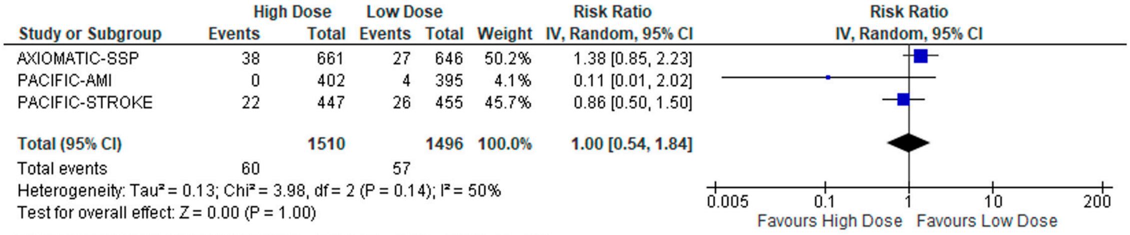

Test for subgroup differences: $Chi^2 = 0.02$, df = 1 (P = 0.89), $I^2 = 0\%$

Figure S29: Forest plot presenting the dose effect of factor XIa inhibitors on the composite of major bleeding or clinically relevant non-major bleeding, with the low dose as the reference dose, after stratification for intermediate and high doses of factor XIa inhibitor.

Study or Subgroup	Intermediate Dose Events	Intermediate Dose Total	Low Dose Events	Low Dose Total	Weight	Risk Ratio IV, Random, 95% CI
AXIOMATIC-SSP	12	325	21	638	23.9%	1.12 [0.56, 2.25]
PACIFIC-AMI	32	397	30	395	50.8%	1.06 [0.66, 1.71]
PACIFIC-STROKE	14	446	19	445	25.3%	0.74 [0.37, 1.45]
**Total (95% CI)**		**1168**		**1478**	**100.0%**	**0.98 [0.70, 1.38]**
Total events	58		70			

Heterogeneity: Tau² = 0.00; Chi² = 0.94, df = 2 (P = 0.62); I² = 0%
Test for overall effect: Z = 0.12 (P = 0.91)

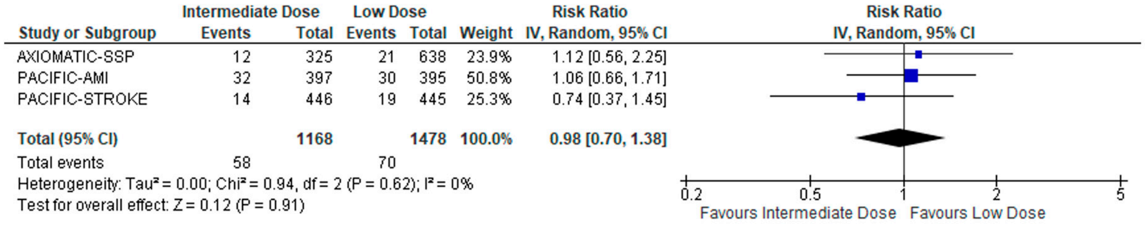

Study or Subgroup	High Dose Events	High Dose Total	Low Dose Events	Low Dose Total	Weight	Risk Ratio IV, Random, 95% CI
AXIOMATIC-SSP	27	650	21	638	29.6%	1.26 [0.72, 2.21]
PACIFIC-AMI	42	402	30	395	46.4%	1.38 [0.88, 2.15]
PACIFIC-STROKE	19	443	19	445	24.0%	1.00 [0.54, 1.87]
**Total (95% CI)**		**1495**		**1478**	**100.0%**	**1.24 [0.92, 1.69]**
Total events	88		70			

Heterogeneity: Tau² = 0.00; Chi² = 0.65, df = 2 (P = 0.72); I² = 0%
Test for overall effect: Z = 1.40 (P = 0.16)

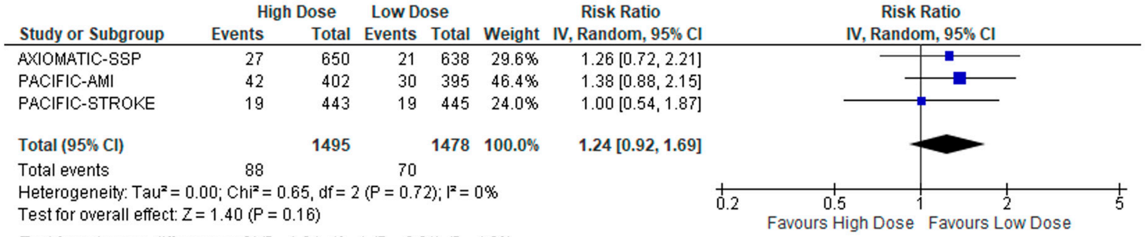

Test for subgroup differences: Chi² = 1.04, df = 1 (P = 0.31), I² = 4.0%
